# Research perspectives on animal health in the era of artificial intelligence

**DOI:** 10.1186/s13567-021-00902-4

**Published:** 2021-03-06

**Authors:** Pauline Ezanno, Sébastien Picault, Gaël Beaunée, Xavier Bailly, Facundo Muñoz, Raphaël Duboz, Hervé Monod, Jean-François Guégan

**Affiliations:** 1 INRAE, Oniris, BIOEPAR, Nantes, France; 2 INRAE, EpiA, Theix, France; 3 ASTRE, Univ Montpellier, CIRAD, INRAE, Montpellier, France; 4 Sorbonne Université, IRD, UMMISCO, Bondy, France; 5 Université Paris-Saclay, INRAE, Jouy-en-Josas, MaIAGE France; 6 MIVEGEC, IRD, CNRS, Univ Montpellier, Montpellier, France; 7 Comité National Français Sur Les Changements Globaux, Paris, France

**Keywords:** Animal disease, Data, Livestock, Modelling, Artificial intelligence, Decision support tool

## Abstract

Leveraging artificial intelligence (AI) approaches in animal health (AH) makes it possible to address highly complex issues such as those encountered in quantitative and predictive epidemiology, animal/human precision-based medicine, or to study host × pathogen interactions. AI may contribute (i) to diagnosis and disease case detection, (ii) to more reliable predictions and reduced errors, (iii) to representing more realistically complex biological systems and rendering computing codes more readable to non-computer scientists, (iv) to speeding-up decisions and improving accuracy in risk analyses, and (v) to better targeted interventions and anticipated negative effects. In turn, challenges in AH may stimulate AI research due to specificity of AH systems, data, constraints, and analytical objectives. Based on a literature review of scientific papers at the interface between AI and AH covering the period 2009–2019, and interviews with French researchers positioned at this interface, the present study explains the main AH areas where various AI approaches are currently mobilised, how it may contribute to renew AH research issues and remove methodological or conceptual barriers. After presenting the possible obstacles and levers, we propose several recommendations to better grasp the challenge represented by the AH/AI interface. With the development of several recent concepts promoting a global and multisectoral perspective in the field of health, AI should contribute to defract the different disciplines in AH towards more transversal and integrative research.

## Introduction

**Figure 1:**
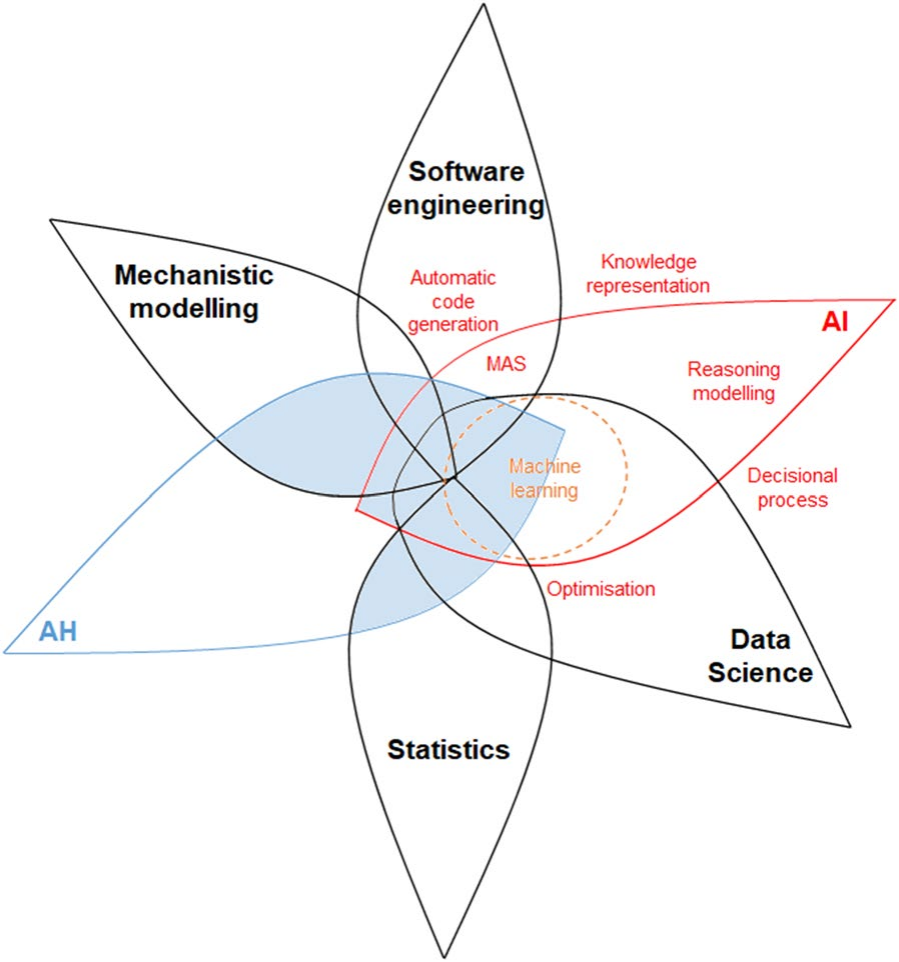
**Interactions between animal health (AH), artificial intelligence (AI), and closely related research domains.** This illustration is pinpointing only the links between AH (in blue), AI and its main subfields (in red), and other related fields of research (in black). It can be naturally complexified through the interactions between AH and other research topics (e.g., human medicine) or between core disciplines (e.g., statistics and physics).

Artificial intelligence (AI) encompasses a large range of theories and technologies used to solve problems of high logical or algorithmic complexity. It crosses many disciplines, including mechanistic modelling, software engineering, data science, and statistics (Figure 1). Introduced in the 1950s, many AI methods have been developed or extended recently with the improvement of computer performance. Recent developments have been fuelled by the interfaces created between AI and other disciplines, such as bio-medicine, as well as massive data from different fields, particularly those associated with healthcare [1, 2].

AI addresses three challenges that also make sense in animal health (AH): (1) understanding a situation and its dynamics, e.g., epidemic spread; (2) the perception of the environment, which corresponds in AH to the detection of patterns (e.g., repeated sequence of observations), forms (e.g., of a protein) and signals (e.g., increased mortality compared to a baseline) at different scales; (3) computer-based decision making, or, more realistically, human decision support (e.g., expert systems, diagnostic support, resource allocation).

To answer these challenges, a wide range of concepts and methods are developed in AI. This includes machine learning (ML), a widely known AI method nowadays, which has been developing since the 1980s [3]. Since the 2000s, deep learning is developing with the rise of big data and the continuous increasing of computing capacities, enabling the exploration of massive amount of information that cannot be processed by conventional statistical methods. In addition, this also includes methods and algorithms for solving complex problems, automating tasks or reasoning, integrating information from heterogeneous sources, or decision support (Figure 1). These methods are now uprising in the human health sector, but are still rarely used to study animal health issues that they would help to revisit.

Part of the scientific challenges faced in AH can be approached from a new perspective by using some of these AI methods to analyse the ever-increasing data collected on animals, pathogens, and their environment. AH research benefits from advances in machine and deep learning methods, e.g., in predictive epidemiology, individual-based precision medicine, and to study host–pathogen interactions [2, 4]. These methods contribute to disease diagnosis and individual case detection, to more reliable predictions and reduced errors, to speed-up decisions and improved accuracy in risk analysis, and to better targeting interventions in AH [5]. AH research also benefits from scientific advances in other domains of AI. Knowledge representation and modelling of reasoning [6] allow more realistic representations of complex socio-biological systems such as those encountered in AH. Examples include processes related to decision-making and uncertainty management [7, 8], as well as of patient life courses like in human epidemiology [9]. This contributes to making them more readable by noncomputer experts. In addition, advances in problem solving under constrained resource allocation [10], in autonomous agents [11], multi-agent systems [12], and multi-level systems [13], as well as on automatic computer code generation [14] can be mobilised to enhance efficient and reliable epidemiological models. Interestingly, this may aid to anticipate the effect of control and management decisions at different spatial and temporal scales (animal, herd, country…).

Conducting research at the AH/AI interface also leads to identify new challenges for AI, on themes common with human health but considering different contexts and perspectives [15]. First, taking into account the particular agro- and socio-economic conditions of production systems is crucial when dealing with AH. Animal production systems depend on human activities and decisions. They can be a source of income (e.g., livestock) or labour forces and source of food in family farming. Citizens have also high expectations in terms of ethics and animal welfare [16]. Conventional measures to control animal diseases may no longer be acceptable by society (e.g., mass culling during outbreaks [17], antimicrobial usage, [18]). Alternatives must be identified and assessed, and AI can contribute. For example, individual-based veterinarian medicine is emerging, mobilising both AI methods and new AH data streams, these data differing from data in human health [19]. The integration of data from deep sequencing in AH, including emerging technologies for studying the metabolome and epigenome, is also a challenge [20, 21]. Second, interactions between animal species, in particular between domestic animals and wildlife, lead to specific infectious disease risks (e.g., multi-host pathogens such as for African swine fever, pathogens crossing the species barrier facilitated by frequent contacts and promiscuity). The intensity of such interactions could increase due to separate or synergistic actions of environmental (e.g., landscape homogenisation, land use change for agriculture development, climate change), demographic (e.g., increasing global demand for animal production) and societal (e.g., outdoor livestock management) pressures. In addition, working on multi-species disease networks provides crucial information on the underlying molecular mechanisms favouring interspecific transmission [22]. Third, animal populations are governed by recurrent decision-making that also impacts health management (e.g., trade, control measures). Economic criteria as consequences on livestock farmers’ incomes are therefore essential indicators for evaluating AH control strategies, which can sometimes be misunderstood or may be at odds with societal expectations. These specificities make the AH/AI interface a theme of interest to stimulate new methodological work and to solve some of old and current locks faced by AH research today. With the development of new concepts in health such as One Health, Ecohealth and Planetary Health, promoting multidisciplinarity, stakeholders’ participation, data sharing, and tackling the complexity of health issues (e.g., multi-host pathogen transmission, short and long-term climatic impacts on disease patterns [23]), AI could participate in this new development by making it possible to technically solve some of the complex problems posed.

Mobilising the literature published at the AH/AI interface between 2009 and 2019 (Additional file 1A), focusing our literature search on mainly livestock and wildlife, as well as interviews conducted with French researchers positioned at this interface (Additional file 1B), we identified the main research areas in AH in which AI is currently involved country-wide. We explored how AI methods contribute to revisiting AH questions and may help remove methodological or conceptual barriers within the field. We also analysed how AH questions interrogate and stimulate new AI technical or scientific developments. In this paper, we first discuss issues related to data collection, organisation and access (Section 1), then we discuss how AI methods contribute to revisiting our understanding of animal epidemiological systems (Section 2), to improving case detection and diagnosis at different scales (Section 3), and to anticipating pathogen spread and control in a wide range of scenarios in order to improve AH management, facilitate decision-making and stimulate innovation (Section 4). Finally, we present the possible obstacles and levers to the development of AI in modern AH (Section 5), before making recommendations to best address the new challenges represented by this AH/IA interface (Section 6).

## Collect, organise and make accessible quality data

A central point for research in AH remains the quality and availability of data, at the different organization levels of living systems and therefore at different spatial and temporal scales [24]. Data of interest are diverse. They can be obtained thanks to molecular analysis (e.g., genomic, metagenomics, or metabolic data), from observational data on individuals (e.g., body temperature, behaviour, milk production and composition, weight, feed intake), or from the production system (e.g., herd structure, breeding, management of sanitary issues). They can also be obtained at larger scales, beyond herds or local groups of animals (e.g., epidemiological data, demographic events, commercial movements, meteorological data, land-use occupation).

Even though the acquisition of these massive and heterogeneous data remain challenging (e.g., metabolome data), a large and diverse amount is already collected: (i) through mandatory reporting in accordance with regulations (e.g., commercial movements of cattle, epidemio-surveillance platform), (ii) by automatic devices (e.g., sensors, video surveillance systems), and (iii) on an ad hoc basis as part of research programs. This leads to a very wide diversity of data properties, and therefore of their management, access and possible uses. These data can be specifically obtained for certain animals or herds (e.g., during cohort monitoring programs) or by private companies (e.g., pig trade movements such as in France, milk collection). This can limit accessibility to academics and public research. Globalisation and large-scale animal trade may generate the need to use data obtained at worldwide scale in AH, especially for quantitative epidemiology (e.g., transcontinental spread of pathogens, animal genetics and breed management) leading to standardisation issues [25].

Consideration should be given to future systems for observing, collecting and managing these data [26], and to practices aimed at better collaboration between stakeholders. While data management has always been an important element in applied research to facilitate their use and valorisation, it is now a strategic issue both in theoretical and more applied research, coupled with a technical and algorithmic challenge [27–29]. Indeed, producing algorithms to manage massive data flows and stocks, by optimising calculations, is a challenge, particularly in real time. It seems also necessary to make heterogeneous data sources interoperable, requiring dedicated methodological developments [25]. In addition, much of the data is private, with ownership often heterogeneous (e.g., multiple owners, non-centralised data, closed data) and sometimes unclear (e.g., lack of knowledge of the real owner of the data between, for example, farmers, the data collector or the farmers’ union). All this tends to considerably complicate access to the data, raises questions about intellectual property, and raises questions in relation to regulations with regards to data protection, e.g., the adaptation of regulation to AH while respecting the confidentiality of the personal data mobilised. What is the relevant business model for data collection or access to existing databases? What about the openness of AH data (e.g., duality between the notion of public good and the private nature of certain data) to make it possible to experiment in real situations and compare the performance of AI algorithms? Answering these questions would facilitate the collection and sharing of ad hoc data. AI, particularly when combining a participatory framework with expert systems and multi-agent systems, helps to build realistic representations of complex socio-biological systems. Thus, it proves to be an effective tool to promote the collaboration of different stakeholders in collective and optimised decision-making, and to assess of the impact of changes in uses and practices [30].

Encouraging experimentation of AI technologies at a territorial scale becomes crucial to favour their development, validate their performance, and measure their predictive quality. In AH, simplified access to data-generating facilities would allow innovative solutions to be tested on a larger scale and would accelerate their development and evaluation. Substantial expertise exists (e.g., epidemiological data platform, large cohorts, experimental farms) that could be put to good use. In addition, AI could help to revisit sampling methods for field data collection in AH and epidemiological surveillance, by better and more dynamically targeting the data to be collected while avoiding redundant collinear, non-necessary data.

## Contribution of AI to better understand animal epidemiological systems

Recent technological advances involving AI approaches have made it possible to obtain vast quantities of measurements and observations, as well as to store and share these data more efficiently. This has resulted in an increasing requirement for appropriate data analytical methods. AI methods emerged as the response of the computer-science community to these requirements, leveraging the exponential improvements in computational power. In parallel, statistical methods have greatly evolved in the last few decades as well, e.g., with regards to dimensionality-reduction in the spaces of variables and parameters, variable selection, and model comparison and combination. The rise in computational power has unleashed the development of Bayesian inference through simulation or approximate methods [31]. Bayesian methods have, in turn, facilitated the integration of data from diverse sources, the incorporation of prior knowledge and allowed for inference on more complex and realistic models while changing the paradigm of statistical inference [32–34].

### Better understanding the evolution of AH and socio-ecological systems in a One Health context

Learning methods can be used to do phylogenetic reconstructions, contributing in particular to new evolutionary scenarios of pathogens and their transmission pathways. For example, phylogenetic models offer an interesting perspective for identifying environmental bacterial strains with high infectious potentiality [35], or for predicting the existence of putative host reservoirs or vectors [36]. The analysis of pathogen sharing among hosts has been used to classify the potential reservoirs of zoonotic diseases using machine learning [37]. The analysis of pathogen genomes can also be used to identify genotypes of animal pathogens that are more likely to infect humans [38].

Using phenomenological niche models that rely on data distribution more than on hypotheses about ecological processes at play, disease occurrence data or retrospective serological data coupled with environmental variables can be related to the risk of being exposed to a pathogen. Thus, they can help monitor potential spillovers and emerging risks and anticipate the epidemic pathogen spread [39]. For instance, Artificial Neural Networks (ANN) have identified the level of genetic introgression between wild and domesticated animal populations in a spatialized context [40], which may help to understand gene diffusion in host × pathogen systems involving multiple host species, and characterise specimen pools at higher risk to act as pathogen spreaders or sinks. Other AI approaches such as multi-agent models, a more mechanistic approach, have been used in an explicit spatial context for vector-borne pathogen transmissions, and proved to be sufficiently versatile to be adapted to several other particular contexts [12].

It should be noted here that several studies reveal the relatively ancient nature of AI research in AH. Such AI methods have often made it possible to identify signals (e.g., genetic introgression) or even particular patterns or properties (e.g., importance of density-dependence in the vector-borne transmission) that are less visible or hardly detectable by more conventional statistical treatments.

All these approaches contribute to better understand pathogen transmission in complex system networks as generally observed for emerging infections in tropical, developing regions of the world. On this matter, an improved knowledge is key for protecting humans against these new threats, and AI/AH interfaces development and training in cooperation with the poorest countries would facilitate synergistic effects and actions to predict and tackle new disease threats.

### Reliability, reproducibility and flexibility of mechanistic models in AH

Better understanding and predicting pathogen spread often requires an explicit and integrative representation of the mechanisms involved in the dynamics of AH systems, irrespective of the scale (within-host: [41]; along a primary production chain: [42]; in a territory: [43, 44]; over a continent: [45]).

Mathematical (equations) or computer-based (simulations) models can be used. Such mechanistic models (i.e., which represent the mechanisms involved in the infection dynamics), when sufficiently modular to represent contrasted situations, make it possible to anticipate the effects of conventional but also innovative control measures (e.g., new candidate molecules, sensors, genomic selection; [46]).

However, to assess realistic control measures, mechanistic epidemiological models require the integration of observational data and knowledge from biology, epidemiology, evolution, ecology, agronomy, sociology or economics. Their development can rapidly face challenges of reliability, transparency, reproducibility, and usage flexibility. Moreover, these models are often developed de novo, making little use of previous models from other systems. Finally, these models, even based on realistic biological hypotheses, may be considered negatively as black boxes by end users (health managers), because the underlying assumptions often became hidden in the code or equations.

The integration of multiple modelling perspectives (e.g., disciplines, points of view, spatio-temporal scales) is an important question in the modelling-simulation field. Epidemiological modelling could benefit from existing tools and methods developed in this field [47–49]. Although essential, good programming practices alone [50] cannot meet these challenges [51]. Scientific libraries and platforms accelerate the implementation of the complex models often needed in AH. For example, the *R* library SimInf [52] helps integrate observational data into mechanistic models. The BROADWICK framework [53] provides reusable software components for several scales and modelling paradigms, but still requires modellers to write large amounts of computer code.

New methods at the crossroads between software engineering and AI can enhance transparency and reproducibility in mechanistic modelling, fostering communication between software scientists, modellers and AH researchers throughout the modelling process (e.g., assumption formulation, assessment, and revision). Knowledge representation methods from symbolic AI, formalised using advanced software engineering methods such as domain-specific languages (DSL, e.g., in KENDRICK for differential equation models: [54]), makes model components accessible in a readable structured text file instead of computer code. Hence, scientists from various disciplines and field managers can be more involved in the model design and evaluation. Scenario exploration and model revision also no longer require rewriting the model code.

**Figure 2:**
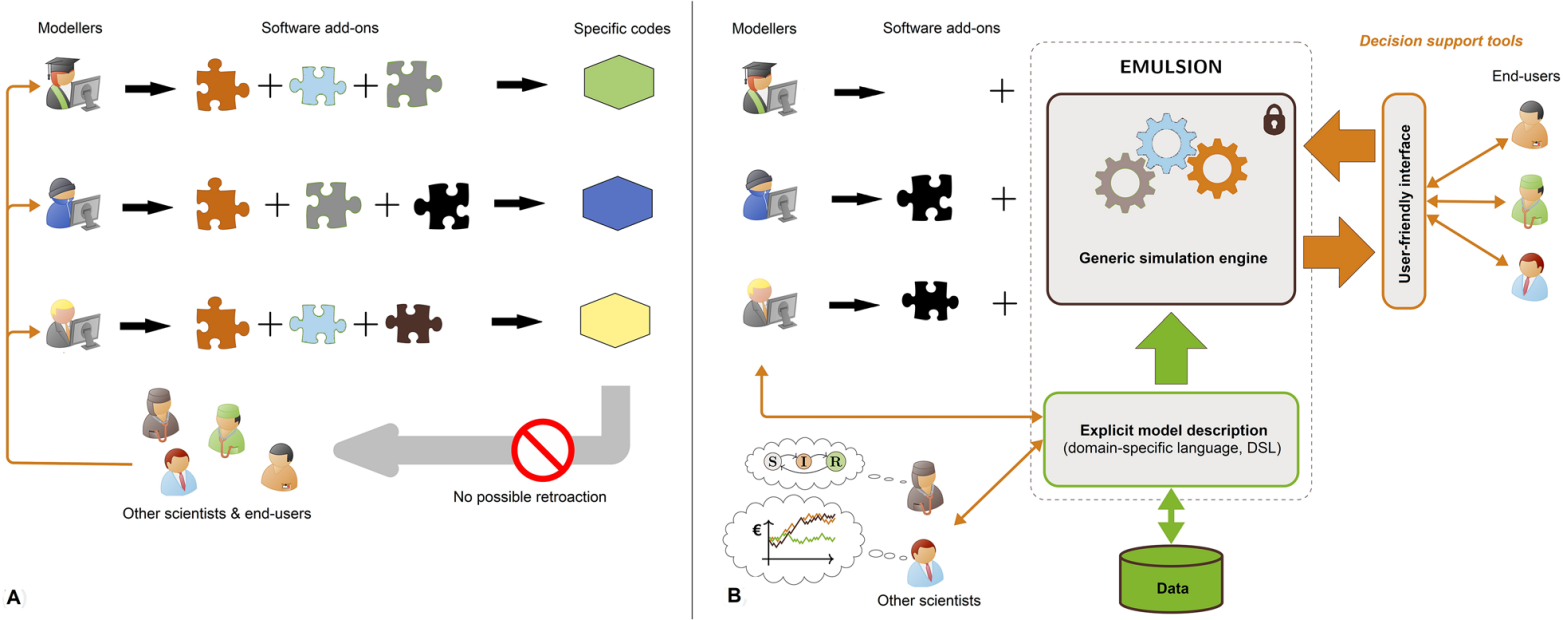
**AI at the service of mechanistic epidemiological modelling** **(adapted from** [51]**)**. **A**. Modellers develop each epidemiological model de novo, producing specific codes not easily readable by scientists from other disciplines or by model end-users. **B**. Using AI approaches to combine a domain-specific language and an agent-based software architecture enhances reproducibility, transparency, and flexibility of epidemiological models. A simulation engine reads text files describing the system to automatically produce the model code. Complementary add-ons can be added if required. Models are easier to transfer to animal health managers as decision support tools.

Other AI methods can improve model flexibility and modularity. Autonomous software agents enable to represent various levels of abstraction and organisation [55], helping modellers go more easily back and forth within small and larger scales, and ensure that all relevant mechanisms are adequately formalised at proper scales (i.e., scale-dependency of determinants and drivers in hierarchical living systems). Combining knowledge representation (through a DSL) and such a multi-level agent-based simulation architecture (e.g., in EMULSION, Figure 2, [56]) enables to encompass several types of models (e.g., compartmental, individual-based) and scales (e.g., individual, population, territory), and it tackles simultaneously the recurring needs for transparency, reliability and flexibility in modelling contagious diseases. This approach should also facilitate in the future the production of support decision tools for veterinary and public health managers and stakeholders.

### Extracting knowledge from massive data in basic AH biology

Supervised, unsupervised and semi-supervised learning methods facilitate basic research development in biology and biomedicine, for example by using morphological analyses to study cell mobility [57]. The use of classification approaches and smart filters allows nowadays to sort massive molecular data (e.g., data from high throughput sequencing and metagenomics). Metabolic, physiological and immunological signalling pathways are explored, and metabolites are identified and quantified in complex biological mixtures, which was before a major challenge [58]. In addition, diagnostic time may be reduced by developing image analysis processing (e.g., accelerated detection of clinical patterns; [59, 60]), often necessary to study host–pathogen interactions in animal pathology. For example, the use of optimisation methods has improved the understanding of the fragmentation of prion assemblages, contributing to a significant reduction in the time required to diagnose neurodegenerative animal diseases, thus paving the way for identifying potential therapeutic targets [61]. In livestock breeding, there is a methodological transition underway from traditional prediction strategies to more advanced machine learning approaches including artificial neural networks, deep learning and Bayesian networks which are being used to improve the reliability of genetic predictions and further the understanding of phenotypes biology. [62].

In human health, new disciplines have emerged in the second half of the 20^th^ century at the interface between AI and flagship disciplines, such as cell biology and immunology. Interface disciplines have developed, e.g., computational biology and immunology, which today must spread to AH. Current human immunology is based on the description of fine molecular and cellular mechanisms (e.g., the number of known interleukins has increased considerably compared to the 1970s). The desire to understand the processes underlying immune responses has led to a revolution by inviting this discipline to focus on complex systems biology and AI-based approaches [63]. However, the imbalance between the numbers of immunologists and immunology modellers is hampering the fantastic growth of this new discipline.

As an additional level of complexity, the hierarchical nature of biological systems makes that at the individual level, animals including humans must be considered as holobionts made of myriads of hosted microbial forms that form discrete ecological units (i.e., infracommunities). The potential of AI to grasp such diversity and complexity (e.g., tissue-specific microbiotes) and to scaling-up to higher levels of organization (e.g., component and compound communities of microbes, including pathogens, circulating in herd and in a given region) is certainly tremendous and should be studied with the same vigour as recent development in computational biology and immunology [40].

## Revisiting AH case detection methods at different scales

**Figure 3:**
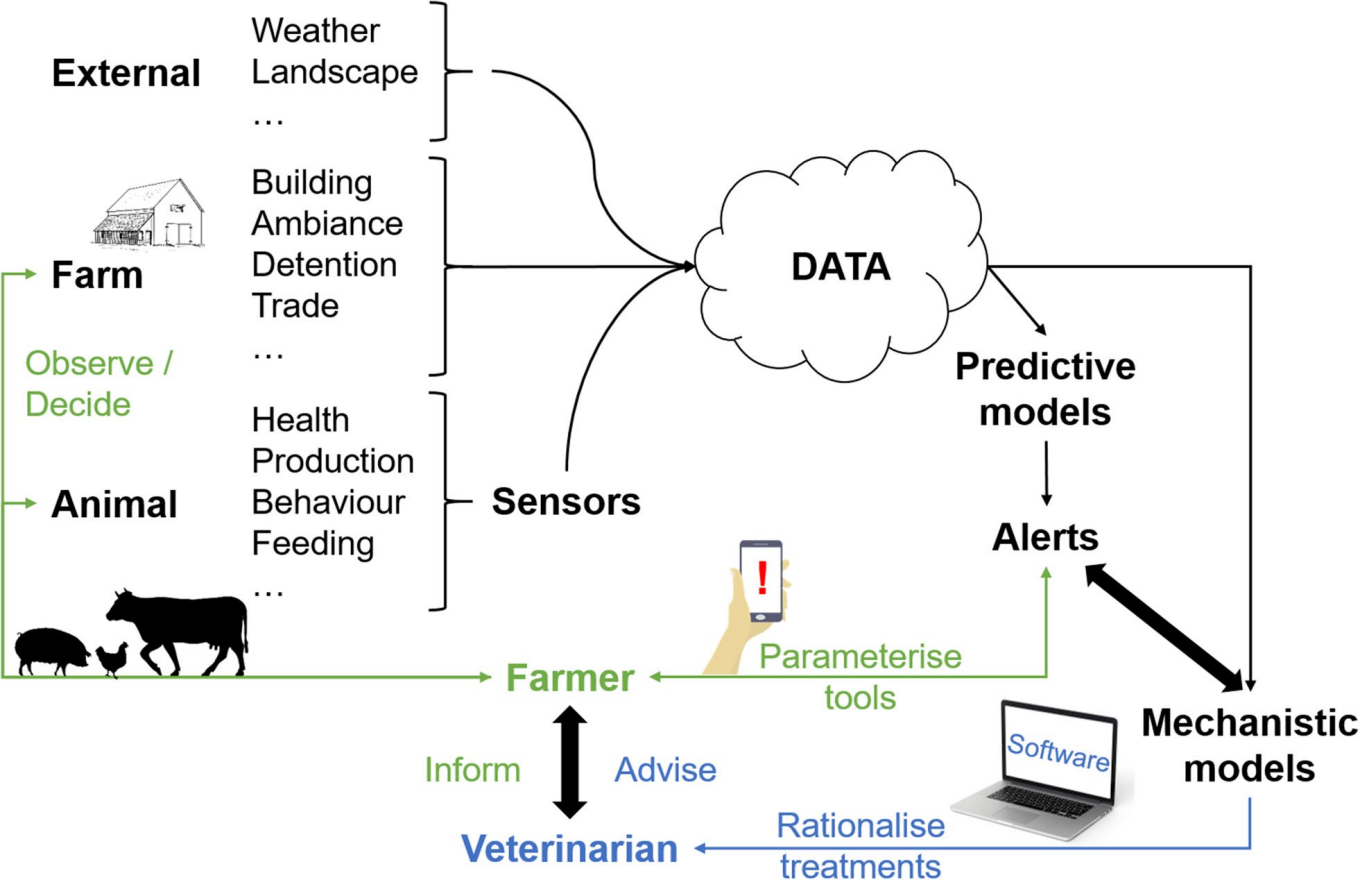
**Extracting information from massive data to monitor animal health and better rationalise treatments.**

Managing livestock health issues requires effective case detection methods, at the individual or even infra-individual (organ) scale, at the group/herd scale, or at larger scales (e.g., territories, countries). Machine learning methods allow detecting patterns and signals in massive data, e.g., in spatial data or time-series of health syndromes and disease cases, contributing to the development of smart agriculture and telemedicine (Figure 3). Alerts can be produced, and contribute to management advice in numerical agriculture [64] and veterinary practices [65]. AI may contribute to an earlier detection of infected cases and the rationalisation of treatments (including antimicrobials) in farm animals, by analysing data collected from connected sensors [66], by targeting individuals or groups of animals [59], or even by using mechanistic models to predict the occurrence of case detections and their treatment [67]. Also, machine learning methods enable to discriminate pathogen strains and thus to better understand their respective transmission pathways if different [68]. Finally, therapeutic strategies can be reasoned through multi-criteria optimisation, by identifying whom to treat in a herd, when, according to what protocol and for how long, in order to maximise the probability of cure while minimising both the risk of drug resistance and the volume or number of doses that are necessary (i.e., individual-based and precision medicine).

Nevertheless, alert quality depends on the quality and representativeness of the datasets used by the learning algorithms. Numerous biases (e.g., hardware, software, human) can affect prediction accuracy. Moreover, alerts produced after training necessarily reflect the specificities of the system from which the data originates (e.g., area, period, rearing practices). Thus, result transposition to other epidemiological systems or to the same system subjected to environmental or regulatory changes remains risky. Furthermore, while machine learning methods (e.g., classification, image analysis, pattern recognition, data mining) provide solutions for a wide range of biomedical and bio-health research questions, it is crucial to demonstrate the performance of these methods by measuring their predictive quality and comparing them to alternative statistical methods whenever possible [69].

At the population level, case detection is based on direct (detection of syndromes) or indirect surveillance, mobilising syndrome proxies. Hence, the emergence of some animal diseases can be detected by syndromic surveillance, by detecting abnormal or rare signals in routine data (e.g., mortality, reproduction, abortion, behaviour, milk production, increased drug use; [70]). Also, serological data can be used retrospectively to identify individual characteristics related to a risk of being exposed to a pathogen, and thus orientate management efforts (e.g., in wildlife; [71]). Statistics and AI are largely complementary to address such issues. Both mobilise the wide range of available data, which are highly heterogeneous, massive and mostly sparse, to detect signals that are often weak or scarce [28, 72, 73]. Such signals can be proxy records (e.g., emergence of infectious diseases following environmental disturbances), health symptoms and syndromes, or even metabolic pathways in cascades which can be precursors of chronic or degenerative diseases. AI also includes methods to mobilise information available on the web. For example, semi-automatic data mining methods enabled to identify emerging signals for international surveillance of epizooties [74] or to analyse veterinary documents such as necropsy reports [75, 76]. Methods from the field of natural language processing can compensate the scarcity of data by extracting syntactic and semantic information from textual records, triggering alerts on new emerging threats that could have been missed otherwise.

On a large to very large scale (i.e., territory, country, continent, global), data analysis of commercial animal movements between farms makes it possible to predict the associated epidemic risk [77, 78]. These movements are difficult to predict, particularly since animal trade is based on many factors associated with human activities and decisions. Methods for recognising spatio-temporal patterns and methodological developments for the analysis of oriented and weighted dynamic relational graphs are required in this field because very few of the existing methods allow large-scale systems to be studied, whereas datasets are often very large (e.g., several tens or even hundreds of thousands of interacting operations).

On this topic, the specific frontier between learning methods of AI and statistics is relatively blurred, lying most on the relative prominence of the computational performance of algorithms versus mathematics, probability and rigorous statistical inference. While machine learning methods are more empirical, focused on improving their predictive performance, statistics is more concerned with the quantification and modelling of uncertainties and errors [79, 80]. In the last decade, both communities have started to communicate and to mix together. Methods have cross-fertilised, giving birth to statistical models using synthetic variables generated by AI methods, or AI algorithms optimising statistical measures of likelihood or quality. New research areas, such as Probabilistic Machine Learning, have emerged at the interface between the two domains [1, 80, 81]. Meanwhile, machine learning and statistics have kept their specific interests and complementarity; machine learning methods are especially well-suited to processing non-standard data types (e.g., images, sounds), while statistics can draw inference and model processes for which only few data are available, or where the quantities of interest are extreme events.

## Targeted interventions, model of human decisions, and support of AH decisions

### Choosing among alternatives

A challenge for animal health managers is to identify the most relevant combinations of control measures according to local (e.g., farm characteristics, production objectives) and territorial (e.g., available resources, farm location, management priorities) specificities. They have to anticipate the effects of health, environmental and regulatory changes, and deliver quality health advice. The question also arises of how to promote innovation in AH, such as to anticipate the required characteristics of candidate molecules in vaccine strategies or drug delivery [82, 83], or to assess the competitive advantage of new strategies (e.g., genomic selection of resistant animals, new vaccines) over more conventional ones. Private (e.g., farmers, farmers’ advisors) and collective managers (e.g., farmer groups, public authorities) need support decision tools to better target public incentives, identify investments to be favoured by farmers [46] and target the measures as effectively as possible: who to target (which farms, which animals)?; with which appropriate measure(s)?; when and for how long? These questions become essential to reasoning about input usage (e.g., antimicrobials, pesticides, biocides) within the framework of the agro-ecological transition.

**Figure 4:**
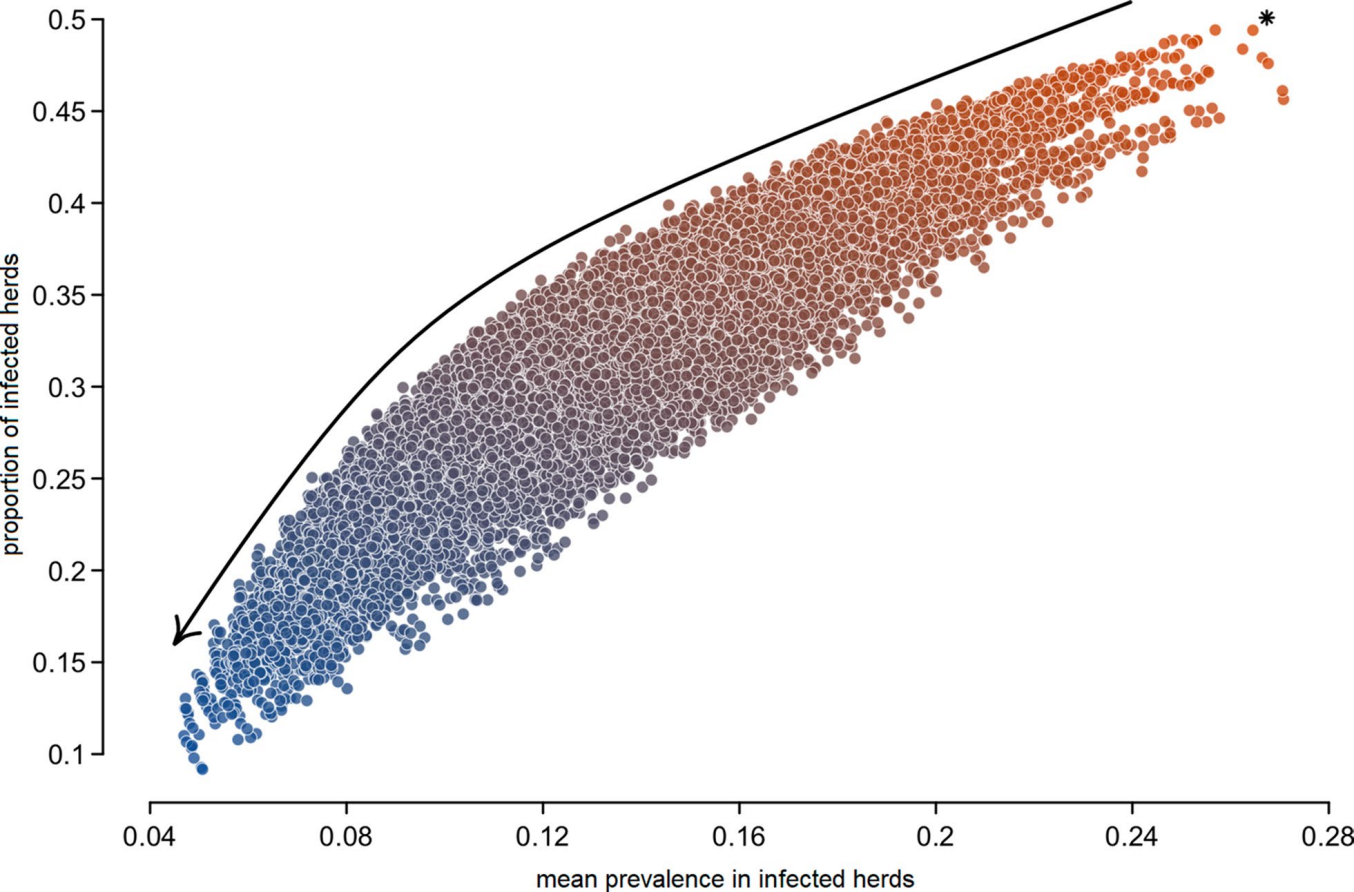
**Identifying relevant strategies to control bovine paratuberculosis at a regional scale** **(adapted from** [76]**)**. Classically, identifying relevant strategies means defining them a priori and comparing them, e.g., by modelling. Only a small number of alternatives can be considered. If all alternatives are considered as in the figure, it results in a multitude of scenarios whose analysis becomes challenging. Here, each point corresponds to the epidemiological situation after 9 years of pathogen spread over a network of 12 500 dairy cattle herds for a given strategy (asterisk: no control). Initially, 10% of the animals are infected on average in 30% of the herds. The blue dots correspond to the most favourable strategies. Mobilizing AI approaches in such a framework, especially optimization under constraints, would facilitate the identification of relevant strategies by exploring the space of possibilities in a more targeted manner.

The use of mechanistic modelling is a solution to assess, compare and prioritise ex ante a wide range of options (Figure 4; [84]). However, most of the available models do not explicitly integrate human decision-making, while control decisions are often made by farmers (e.g., unregulated diseases), with sometimes large-scale health and decision-making consequences (e.g., pathogen spread, dissemination of information and rumours, area of influence). Recent work aims to integrate humans and their decisions by mobilising optimal control and adaptive strategies from AI [7, 85] or health economics methods [86, 87]. A challenge is to propose clear and context-adapted control policies [88]. Such research is just starting in AH [46] and must be extended as part of the development of agro-ecology, facing current societal demand for product quality and respect for ecosystems and their biodiversity on one side, animal well-being and ethics on the other side, and more generally international health security.

### Accounting for expectations and fears of animal health managers

Animal health managers should have access to model predictions in a time frame compatible with management needs, which is problematic in the face of unpredictable emerging events (e.g., new epidemiological systems, transmission pathways, trade patterns, control measures). Developing a library of models included in a common framework would strengthen the responsiveness of modellers in animal epidemiology. Relevant models would be developed more quickly and would gain accuracy from real-time modelling as epidemics progress [89, 90]. However, if this makes move more quickly from concepts (knowledge and assumptions) to simulations and support decision tools, a gain in performance is still required to perform analyses at a very large scale. The automatic generation of high-performance computer code could be a relevant solution, which however remains a crucial methodological lock to be addressed in AI. In addition, it is often required to perform a very large number of calculations or to analyse very large datasets, which call for a rational use of computing resources. Software transferred to health managers sometimes require the use of private cloud resources (i.e., it does not run on simple individual computers), highlighting the trade-offs between simulation cost, service continuity (e.g., failure management) and time required to obtain simulation results [91]. These questions are currently related to computer science research, and collaborations are desirable between these researchers and those from AH.

Managers also wish to rely on accurate predictions from realistic representations of the biological systems. Before being used, model behaviour should be analysed, which raises the questions of exploring the space of uncertainties and data, and of optimization under constraints. This often requires intensive simulations, which would benefit from optimization algorithms to explore more efficiently the space of possibilities. In turn, this would allow, for example, the automatic identification of how to achieve a targeted objective (e.g., reducing the prevalence of a disease below an acceptable threshold) while being constrained in resource allocation. While this issue finds solutions in modern statistics for relatively simple systems, it represents a science front for complex systems (e.g., large scale, multi-host/multi-pathogen systems) that are becoming the norm. In addition, optimization goals specific to AH may generate ad hoc methodological needs [92]. The needs in abstraction and analysis capacity are massive and could benefit from complementarities between AI (e.g., reasoned exploration, intelligent use of computer resources, optimized calculations) and statistics to extract as much information as possible from the data: (1) explore, analyse, predict; (2) infer processes and emergent properties. Methodological developments are still required and would benefit many health issues, particularly in relation to the currently evolving concepts of reservoir-host, edge-host and species barrier [93]. Furthermore, methodological developments and dissemination of existing methods should be reinforced.

Finally, three barriers have been identified to the development of support decision tools for health managers, related to the societal issue of the acceptability of AI sensu lato, as a major factor of progress. First, ethical issues, which are obvious when it comes to human health, are just as important to consider in AH. Which AI-based tools do we want for modern animal husbandries and trades, and for which objectives? Are these tools not likely to lead to discrimination against farms according to their health status, even when this status cannot be managed by the farmer alone? Second, in AH too, there is a fear that AI-tools may replace human expertise. However, automating does not mean replacing human, his expertise and decision [94], but rather supporting his capacities for abstraction and analysis, accelerating the global process, making predictions more reliable, guiding complementary research. Nevertheless, a significant development of computer resources and equipment is not without impacting the environment in terms of carbon footprint (e.g., energy-intensive servers, recycling of sensors), which must also be accounted for. Third, the very high complexity of analysing results and acculturating end-users with knowledge issued from academic research, particularly AI, is an obstacle to the appropriation of AI-tools by their users. This may lead to the preference for simpler and more easily accessible methods. However, the latter may not always be the most relevant or reliable. Citizen science projects, also known as community participation in human epidemiology, enable AH to co-design and co-construct the AI-tools of tomorrow with their end-users [95], to better meet their expectations and needs, and to increase their confidence in the predictions of sometimes obscure research models, especially when they are hard to read (e.g., lines of code). Similarly, these AI-tools could be developed together with public decision-makers, livestock farmers, agro-food industries and sectoral trade unions. Co-construction gives time to explain the science behind the tools and makes it more transparent and useful. This citizen participation, which is nowadays supported in many countries, guarantees decisions more in line with citizens’ expectations and corresponds to a general trend towards structured decision-making. AI must contribute to this democratisation of aid in public decision-making in AH.

## Barriers to the development of research at the AI/AH interface

Research conducted at the interface between AI and AH requires strong interactions between biological disciplines (e.g., infectiology, immunology, clinical sciences, genetics, ecology, evolution, epidemiology, animal and veterinary sciences) and more theoretical disciplines (e.g., modelling, statistics, computer science), sometimes together with sociology and economics. Conducting research at this interface requires strengthening the few teams already positioned in Western Europe, but also bringing together teams working around the concepts of One Health, Ecohealth and Planetary Health to benefit from recent achievements in infectious disease ecology and modelling, plant health and environmental health [96]. This work must be based on a wide range of methodological skills (e.g., learning methods, data mining, information systems, knowledge representation, multi-agent systems, problem solving, metamodeling, optimisation, simulation architecture, model reduction, decision models). The need for research, training and support are crucial issues at national, European and international levels. Also, a facilitated and trusted connection is required between academics, technical institutes, and private partners, who are often the holders or collectors of data of interest to solve AH research questions through AI approaches. The construction of better inter-sectoral communication and coordination must be done at supra-institutional level, as this theme seems hyper-competitive and as some current divisions still go against information and data sharing.

An acculturation of researchers to AI, its methods and potential developments, but also its limitations, must be proposed to meet the challenges of 21^st^ century agriculture. Indeed, there are obstacles to conducting research on this scientific front. Establishing the new collaborations required between teams conducting methodological work and teams in the fields of application remains difficult given the low number of academic staff on these issues, their very high current mobilisation and their low availability to collaborate on new subjects, as well as the difficulty of understanding and mastering these methods. There is a need for watching and training on AI methods available or under development, new softwares/packages, and their applicability. To develop key collaborations and establish a strategic positioning, an interconnection can also be made via transversal teams which appears as a preferential path. Solutions must also be found to encourage method percolation in the community and the development of scientific and engineering skills.

Finally, AI methods, such as classification, machine learning, data mining, and the innovations in AH to which these methods can lead are rarely discussed in veterinary high school education, whereas these students represent the future professionals of AH [96]. Similarly, there is a quasi-absence of sentinel networks of veterinarians, even if it is developing, although AH questions can arise on a large and collective scale. The scientific community would also benefit from further increasing its skills and experience in the valuation, transfer and protection of intellectual property on these AI methods and associated outcomes.

## Levers to create a fruitful AI/AH interface

### Data sharing and protection

No innovation at the interface between AI and AH is possible without strong support for the organisation of data storage, management, analysis, calculation, and restitution. The major risk is that demands for AI developments inflate without being supported by available human resources. In addition, an expertise in law, jurisdiction and ethics is required with regard to the acquisition, holding, use and protection of data in AH. This question must be considered at least at the inter-institutional/national level, and could benefit from a similar thinking already engaged in human health. The issue is to be able to support any change with regard to data traceability to their ownership, whether being from public or private domains.

New data are rich and must be valued as much as possible, not by each owner separately, but through data sharing and the mobilisation of multi-disciplinary skills to analyse such heterogeneous and complex data. Hence, data interoperability skills are required and must be developed. Models for making federated data sustainable over decades are required [97]. In addition, further encouraging the publication of data papers as valuable research products can help to develop the necessary culture of sharing, documentation and metadata.

Finally, to be able to launch ambitious experiments with AI methods on real data, it is necessary to (1) remove unauthorised access to data by negotiating with owners at large scale; (2) analyse and understand the related effect on methodological developments; and (3) if necessary, extend such initiatives to other areas, at national scale, or even across European countries.

### Attract the necessary skills

An undeniable barrier to conduct such research comes from human resources, in particular the current insufficient capacity of supervision by permanent scientists. Collaborations are a solution to attract new skills. However, initiating collaborations at the AH/AI interface becomes very complicated because the qualified teams are already overwhelmed. Skill development at this interface must be supported, the cross-fertilisation of disciplines being essential. A watch on methods must also be carried out, accompanied by explanations for application fields, to train researchers and engineers. Financial incentives for scientist internships in specialised laboratories would increase skill capitalisation in advanced methods, while facilitating future national or international collaborations. In a context of limited resources as observed in many countries nowadays (e.g., new opened positions in national institutions) and limited experts pool (e.g., skills), facilitating post-doctoral fellows and continuing education of researchers becomes crucial. Finally, to consolidate the pool of future researchers in AH, promoting basic AI education in initial training of AH researchers, engineers and veterinarians is paramount.

More specifically concerning current research in immunology, cell biology and infectiology, the contribution of AI has been more widely considered in human health, which could feed a similar reflection in AH as locks and advances are not very specific. Before embarking on the fronts of science (e.g., emerging epigenomics and metabolomics in AH), a few persons from these biological disciplines should acculturate into AI, or even acquire autonomy in the use of methods [98], which internationally tends to be the trend [63]. This can be done through the sharing of experiences and basic training on existing methods, their advantages and limitations compared to other methods coming from statistics and mathematical modelling.

### Encourage the development of AH/AI projects

Projects at the AH/AI interface, like any interdisciplinary project, must mobilise teams from both groups of disciplines and allow everyone to progress in their own discipline. However, identifying the issues shared between the most relevant disciplines requires a good acculturation of the disciplines between them, as well as an otherness aimed at better understanding each other [95], which is not yet the case at the AH/AI interface.

In terms of funding, European project calls offer interesting opportunities, but a significant imbalance persists between the ability to generate data and analyse complex issues, and the availability of human resources and skills to address such issues through AI methods or other modern methods in statistics, mathematics and computer science. The major international foundations (e.g., Bill and Melinda Gates) can also be mobilised on emerging infectious diseases at the animal/human interface (e.g., characterisation of weak signals, phenologies, emergence precursors), with a more significant methodological value. However, risk-taking is rarely allowed by funding agencies, although it is crucial to initiate interdisciplinary work. Dedicated incentive funding would support projects in their initial phase and make larger projects emerge after consolidation of the necessary disciplinary interactions.

Finally, these projects are generally based on the use of significant computing resources. Thus, research institutes and private partners should contribute in a financial or material way to the shared development of digital infrastructures, data centres, supercomputing centres on a national scale, as well as support recognised open-source software platforms on which a large part of the research conducted is based (e.g., Python, *R*).

### Promoting innovation and public–private partnership

Encouraging public–private partnership would promote a leverage effect on public funding and would make it possible to place AI research and development on a long-term basis in AH. Mapping the highly changing landscape of companies in the AH/AI sector, whether international structures or start-ups, would provide a better understanding of the possible interactions. Similarly, mapping academic deliverables produced at this interface would increase their visibility and highlight their potential for valorisation or transfer. Finally, considering the production of documented algorithms as scientific deliverables, along with publications, would help support this more operational research. More broadly, it would be advisable to initiate a communication and education/acculturation policy around AI and its development in AH (e.g., links with the society, farmers, agricultural unions, public services).

## Conclusion

The use of AI methods (e.g., machine learning, expert systems, analytical technologies) converges today with the collecting of massive and complex data, and allows these fields to develop rapidly. However, it is essential not to perceive massive data and AI as the same trend, because the accumulation of data does not always lead to an improvement in knowledge. Nevertheless, the more data are numerous and representative of working concepts and hypotheses, the more important results can be obtained from AI applications. The underlying ethical, deontological and legal aspects of data ownership, storage, management, sharing and interoperability also require that a reflection be undertaken nationally and internationally in AH to better manage these data of multi-sectoral origin and their various uses. Moreover, while the effort to acquire such data is impressive, the development of AI skills within the AH community remains limited in relation to the needs. Opportunities for collaborations with AI teams are limited because these teams are already in high demand. To ensure that AH researchers are well aware of the opportunities offered by AI, but also of the limits and constraints of AI approaches, a training effort must be provided and generalized. Finally, the current boom in AI now makes it possible to integrate the knowledge and points of view of the many players in the field of animal health and welfare further upstream. However, this requires that AI and its actors accept to deal with the specificity and complexity of AH, which is not a simple library of knowledge that can be digitised to search for sequences or informative signals.

## Supplementary Information

**Additional file 1.** Systematic literature review, interviews, previous publication in French.

## References

[1] Hosny A, Parmar C, Quackenbush J, Schwartz LH, Aerts HJWL (2018). Artificial intelligence in radiology. Nat Rev Cancer.

[2] Karczewski KJ, Snyder MP (2018). Integrative omics for health and disease. Nat Rev Genet.

[3] Murphy KP (2012) Machine learning: a probabilistic perspective. In: Adaptive computation and machine learning series. MIT Press, USA

[4] Zhang W, Chien J, Yong J, Kuang R (2017). Network-based machine learning and graph theory algorithms for precision oncology. NPJ Precis Oncol.

[5] Saria S, Butte A, Sheikh A (2018). Better medicine through machine learning: what’s real, and what’s artificial?. PLoS Med.

[6] Bedi G, Carrillo F, Cecchi GA, Fernández Slezak D, Sigman M, Mota NB, Ribeiro S, Javitt DC, Copelli M, Corcoran CM (2015). Automated analysis of free speech predicts psychosis onset in high-risk youths. Schizophrenia.

[7] Maclachlan MJ, Springborn MR, Fackler PL (2017). Learning about a moving target in resource management: optimal Bayesian disease control. Am J Agri Econ.

[8] Lynn LA (2019). Artificial intelligence systems for complex decision-making in acute care medicine: a review. Patient Saf Surg.

[9] Pinaire J, Azé J, Bringay S, Landais P (2017). Patient healthcare trajectory. An essential monitoring tool: a systematic review. Health Inf Sci Syst.

[10] Vrakas D, Vlahavas IPL (2008) Artificial intelligence for advanced problem solving techniques. Information Science Reference, Hershey, PA, pp. 369

[11] Shakshuki E, Reid M (2015). Multi-agent system applications in healthcare: current technology and future roadmap. Proc Comput Sci.

[12] Roche B, Guégan JF, Bousquet F (2008). Multi-agent systems in epidemiology: a first step for computational biology in the study of vector-borne disease transmission. BMC Bioinform.

[13] Picault S, Huang Y-L, Sicard V, Ezanno P (2017) Enhancing Sustainability of Complex Epidemiological Models through a Generic Multilevel Agent-based Approach. In: Proceedings of the 26^th^ International Joint Conference on Artificial Intelligence (IJCAI). pp. 374–380, AAAI. 10.24963/ijcai.2017/53p

[14] Russell S, Norvig P (2010). Artificial intelligence a modern approach.

[15] Ducrot C, Bed’Hom B, Béringue V, Coulon JB, Fourichon C, Guérin JL, Krebs S, Rainard P, Schwartz-Cornil I, Torny D, Vayssier-Taussat M, Zientara S, Zundel E, Pineau T (2011). Issues and special features of animal health research. Vet Res.

[16] Clark B, Stewart GB, Panzone LA, Kyriazakis I, Frewer LJ (2016). A systematic review of public attitudes, perceptions and behaviours towards production diseases associated with farm animal welfare. J Agric Environ Ethics.

[17] Miguel E, Grosbois V, Caron A, Pople D, Roche B, Donnelly C (2020). A systemic approach to assess the potential and risks of wildlife culling for infectious disease control. Commun Biol.

[18] Hur B, Hardefeldt LY, Verspoor K, Baldwin T, Gilkerson JR (2019). Using natural language processing and VetCompass to understand antimicrobial usage patterns in Australia. Aust Vet J.

[19] Behmann J, Hendriksen K, Mueller U, Buescher W, Pluemer L (2016). Support vector machine and duration-aware conditional random field for identification of spatio-temporal activity patterns by combined indoor positioning and heart rate sensors. Geoinformatica.

[20] Suravajhala P, Kogelman LJA, Kadarmideen HN (2016). Multi-omic data integration and analysis using systems genomics approaches: methods and applications in animal production, health and welfare. Genet Sel Evol.

[21] Goldansaz SA, Guo AC, Sajed T, Steele MA, Plastow GS, Wishart DS (2017). Livestock metabolomics and the livestock metabolome: a systematic review. PLoS One.

[22] Anvar SY, Tucker A, Vinciotti V, Venema A, van Ommen GJ, van der Maarel SM, Raz V (2011). Interspecies translation of disease networks increases robustness and predictive accuracy. PLoS Comput Biol.

[23] Morand S, Guégan J-F, Laurans Y (2020) From One Health to Ecohealth, mapping the incomplete integration of human, animal and environmental health. Iddri, Issue Brief No. 04/20

[24] Ezenwa VO, Prieur-Richard A-H, Roche B, Bailly X, Becquart P, Garcia-Peña GE, Hosseini PR, Keesing F, Rizzoli A, Suzán GA, Vignuzzi M, Vittecoq M, Mills JN, Guégan J-F (2015). Interdisciplinarity and infectious diseases: an Ebola case study. PLoS Pathog.

[25] Van Boeckel TP, Takahashi S, Liao Q, Xing W, Lai S, Hsiao V, Liu F, Zheng Y, Chang Z, Yuan C, Metcalf CJE, Yu H, Grenfell BT (2016). Hand, foot, and mouth disease in China: critical community size and spatial vaccination strategies. Sci Rep.

[26] Holmstrom LK, Beckham TR (2017). Technologies for capturing and analysing animal health data in near real time. Rev Sci Tech.

[27] Neethirajan S (2017). Recent advances in wearable sensors for animal health management. Sens Biosensing Res.

[28] Perez AM, Zeng D, Tseng CJ, Chen H, Whedbee Z, Paton D, Thurmond MC (2009). A web-based system for near real-time surveillance and space-time cluster analysis of foot-and-mouth disease and other animal diseases. Prev Vet Med.

[29] Wilkinson MD, Dumontier M, Aalbersberg IJ, Appleton G, Axton M, Baak A, Blomberg N, Boiten J-W, da Silva Santos LB, Bourne PE, Bouwman J, Brookes AJ, Clark T, Crosas M, Dillo I, Dumon O, Edmunds S, Evelo CT, Finkers R, Gonzalez-Beltran A, Gray AJG, Groth P, Goble C, Grethe JS, Heringa J, 't Hoen PAC, Hooft R, Kuhn T, Kok R, Kok J, (2016). The FAIR guiding principles for scientific data management and stewardship. Sci Data.

[30] Binot A, Duboz R, Promburom P, Phimpraphai W, Cappelle J, Lajaunie C, Goutard FL, Pinyopummintr T, Figuié M, Roger FL (2015). A framework to promote collective action within the One Health community of practice: using participatory modelling to enable interdisciplinary, cross-sectoral and multi-level integration. One Health.

[31] Robert CP (2014). Bayesian computational tools. Annu Rev Stat Appl.

[32] Dunson DB (2001). Commentary: practical advantages of Bayesian analysis of epidemiologic data. Am J Epidemiol.

[33] Uusitalo L (2007). Advantages and challenges of Bayesian networks in environmental modelling. Ecol Model.

[34] Fokoué E (2019). On the ubiquity of the Bayesian paradigm in statistical machine learning and data science. Math Appl.

[35] Bailly X (2017). Hidden Markov phylogenetic models offer an interesting perspective to identify “high risk lineages” of environmental pathogens. Infect Genet Evol.

[36] Babayan SA, Orton RJ, Streicker DG (2018). Predicting reservoir hosts and arthropod vectors from evolutionary signatures in RNA virus genomes. Science.

[37] Wardeh M, Sharkey KJ, Baylis M (2020). Integration of shared-pathogen networks and machine learning reveals the key aspects of zoonoses and predicts mammalian reservoirs. Proc Biol Sci.

[38] Li J, Zhang S, Li B, Hu Y, Kang X-P, Wu X-Y, Huang M-T, Li Y-C, Zhao Z-P, Qin C-F, Jiang T (2020). Machine learning methods for predicting human-adaptive influenza A viruses based on viral nucleotide compositions. Mol Biol Evol.

[39] Peters DPC, McVey DS, Elias EH, Pelzel-McCluskey AM, Derner JD, Burruss ND, Schrader TS, Yao J, Pauszek SJ, Lombard J, Rodriguez LL (2020). Big data-model integration and AI for vector-borne disease prediction. Ecosphere.

[40] Lek S, Guégan J-F (2000) Artificial neuronal networks. In: Application to ecology and evolution. Springer, Berlin. 10.1016/j.it.2016.11.006

[41] Go N, Touzeau S, Islam Z, Belloc C, Doeschl-Wilson A (2019). How to prevent viremia rebound? Evidence from a PRRSv data-supported model of immune response. BMC Syst Biol.

[42] Ferrer Savall J, Bidot C, Leblanc-Maridor M, Belloc C, Touzeau S (2016). Modelling Salmonella transmission among pigs from farm to slaughterhouse: interplay between management variability and epidemiological uncertainty. Intern J Food Microbiol.

[43] Widgren S, Engblom S, Bauer P, Frössling J, Emanuelson U, Lindberg A (2016). Data-driven network modelling of disease transmission using complete population movement data: spread of VTEC O157 in Swedish cattle. Vet Res.

[44] Qi L, Beaunée G, Arnoux S, Dutta BL, Joly A, Vergu E, Ezanno P (2019). Neighbourhood contacts and trade movements drive the regional spread of bovine viral diarrhoea virus (BVDV). Vet Res.

[45] Buhnerkempe MG, Tildesley MJ, Lindström T, Grear DA, Portacci K, Miller RS, Lombard JE, Werkman M, Keeling MJ, Wennergren U, Webb CT (2014). The impact of movements and animal density on continental scale cattle disease outbreaks in the United States. PLoS One.

[46] Ezanno P, Andraud M, Beaunée G, Hoch T, Krebs S, Rault A, Touzeau S, Vergu E, Widgren S (2020). How mechanistic modelling supports decision 1 making for the control of enzootic infectious diseases. Epidemics.

[47] Garira W (2018). A primer on multiscale modelling of infectious disease systems. Infect Dis Model.

[48] Traoré M, Zacharewicz G, Duboz R, Zeigler B (2018). Modeling and simulation framework for value-based healthcare systems. Simulation.

[49] Childs LM, El Moustaid F, Gajewski Z, Kadelka S, Nikin-Beers R, Smith JW, Walker M, Johnson LR (2019). Multi-scale models and data for infectious diseases: a systematic review. PeerJ Preprints.

[50] Sandve GK, Nekrutenko A, Taylor J, Hovig E (2013). Ten simple rules for reproducible computational research. PLoS Comput Biol.

[51] Leek JT, Peng RD (2015). Opinion: reproducible research can still be wrong: adopting a prevention approach. Proc Natl Acad Sci USA.

[52] Widgren S, Bauer P, Eriksson R, Engblom S (2016) SimInf: an R package for data-driven stochastic disease spread simulations. ArXiv160501421 Q-Bio Stat. http://arxiv.org/abs/1605.01421

[53] O’Hare A, Lycett SJ, Doherty TM, Salvador LC, Kao RR (2016). Broadwick: a framework for computational epidemiology. BMC Bioinform.

[54] Bui TMA, Stinckwich S, Ziane M, Roche B, Ho TV (2015) KENDRICK: a domain specific language and platform for mathematical epidemiological modelling. In: proc. IEEE RIVF International Conference on Computing and Communication Technologies, Research, Innovation, and Vision for the Future. pp. 132–7. 10.1109/RIVF.2015.7049888

[55] Mathieu P, Morvan G, Picault S (2018). Multi-level agent-based simulations: four design patterns. Simul Model Pract Theory.

[56] Picault S, Huang Y-L, Sicard V, Arnoux S, Beaunée G, Ezanno P (2019). EMULSION: transparent and flexible multiscale stochastic models in human, animal and plant epidemiology. PLoS Comput Biol.

[57] Sebag AS, Plancade S, Raulet-Tomkiewicz C, Barouki R, Vert J-P, Walter T (2015) Inferring an ontology of single cell motions from high-throughput microscopy data. In: Proc. IEEE International Symposium on Biomedical Imaging, Apr. 2015, New-York, USA, pp. 160–163. 10.1109/ISBI.2015.7163840

[58] Tardivel P, Canlet C, Lefort G, Tremblay-Franco M, Debrauwer L, Concordet D, Servien R (2017). ASICS: an automatic method for identification and quantification of metabolites in complex 1D 1H NMR spectra. Metabolomics.

[59] Dórea FC, Muckle CA, Kelton D, McClure JT, McEwen BJ, McNab WB, Sanchez J, Revie CW (2013). Exploratory analysis of methods for automated classification of laboratory test orders into syndromic groups in veterinary medicine. PLoS One.

[60] Gandia P, Jaudet C, Chatelut E, Concordet D (2017). Population pharmacokinetics of tracers: a new tool for medical imaging?. Clin Pharmacokinet.

[61] Chyba M, Coron J-M, Mileyko Y, Rezaei H (2016) Optimization of prion assemblies fragmentation. In: Proc. IEEE Conference on Decision and Control (CDC), Las Vegas, USA, 6

[62] Nayeri S, Sargolzaei M, Tulpan D (2019). A review of traditional and machine learning methods applied to animal breeding. Anim Health Res Rev.

[63] Bassaganya-Riera J, Hontecillas R (2016) Introduction to computational immunology. In: Bassaganya-Riera J (ed) Computational immunology: models and tools. pp. 1–8

[64] Liakos KG, Busato P, Moshou D, Pearson S, Bochtis D (2018). Machine learning in agriculture: a review. Sensors.

[65] Jones-Diette JS, Dean RS, Cobb M, Brennan ML (2019). Validation of text-mining and content analysis techniques using data collected from veterinary practice management software systems in the UK. Prev Vet Med.

[66] Morota G, Ventura RV, Silva FF, Koyama M, Fernando SC (2018). Big data analytics and precision animal agriculture symposium: machine learning and data mining advance predictive big data analysis in precision animal agriculture. J Anim Sci.

[67] Picault S, Ezanno P, Assié S (2019) Combining early hyperthermia detection with metaphylaxis for reducing antibiotics usage in newly received beef bulls at fattening operations: a simulation-based approach. In: Society of veterinary epidemiology and preventive medicine (SVEPM), pp. 13. Utrecht, The Netherland, 27-30/3/2019

[68] Esener N, Green MJ, Emes RD, Jowett B, Davies PL, Bradley AJ, Dottorini T (2018). Discrimination of contagious and environmental strains of *Streptococcus uberis* in dairy herds by means of mass spectrometry and machine-learning. Sci Rep.

[69] Hepworth PJ, Nefedov AV, Muchnik IB, Morgan KL (2012). Broiler chickens can benefit from machine learning: support vector machine analysis of observational epidemiological data. J R Soc Interface.

[70] Marceau A, Madouasse A, Lehébel A, van Schaik G, Veldhuis A, Van der Stede Y, Fourichon C (2014). Can routinely recorded reproductive events be used as indicators of disease emergence in dairy cattle? An evaluation of 5 indicators during the emergence of bluetongue virus in France in 2007 and 2008. J Dairy Sci.

[71] Fountain-Jones NM, Machado G, Carver S, Packer C, Recamonde-Mendoza M, Craft ME (2019). How to make more from exposure data? An integrated machine learning pipeline to predict pathogen exposure. J Anim Ecol.

[72] Charras-Garrido M, Azizi L, Forbes F, Doyle S, Peyrard N, Abrial D (2013). On the difficulty to delimit disease risk hot spots. Int J Appl Earth Obs.

[73] Forbes F, Charras-Garrido M, Azizi L, Doyle S, Abrial D (2013). Spatial risk mapping for rare disease with hidden Markov fields and variational EM. Annals Appl Stat.

[74] Arsevska E, Valentin S, Rabatel J, de Goër de Hervé J, Falala S, Lancelot R, Roche M (2018). Web monitoring of emerging animal infectious diseases integrated in the French Animal Health Epidemic Intelligence System. PLoS One.

[75] Küker S, Faverjon C, Furrer L, Berezowski J, Posthaus H, Rinaldi F, Vial F (2018). The value of necropsy reports for animal health surveillance. BMC Vet Res.

[76] Bollig N, Clarke L, Elsmo E, Craven M (2020). Machine learning for syndromic surveillance using veterinary necropsy reports. PLoS One.

[77] Hoscheit P, Geeraert S, Beaunée G, Monod H, Gilligan CAG, Filipe J, Vergu E, Moslonka-Lefebvre M (2016). Dynamical network models for cattle trade: towards economy-based epidemic risk assessment. J Complex Netw.

[78] Moslonka-Lefebvre M, Gilligan CA, Monod H, Belloc C, Ezanno P, Filipe JAN, Vergu E (2016). Market analyses of livestock trade networks to inform the prevention of joint economic and epidemiological risks. J R Soc Interface.

[79] Efron B (2020). Prediction, estimation, and attribution. J Am Stat Ass.

[80] Ghahramani Z (2012) Probabilistic modelling, machine learning, and the information revolution. MIT Computer Science and Artificial Intelligence Lab, http://mlg.eng.cam.ac.uk/zoubin/talks/mit12csail.pdf, Accessed 17 Oct 2019

[81] Hastie T, Tibshirani R, Friedman JH (2009) The elements of statistical learning: data mining, inference, and prediction. 2^nd^ edn. Springer Series in Statistics. Springer

[82] Goodswen SJ, Kennedy PJ, Ellis JT (2017). On the application of reverse vaccinology to parasitic diseases: a perspective on feature selection and ranking of vaccine candidates. Int J Parasitol.

[83] Schneider G (2019). Mind and machine in drug design. Nat Mach Intell.

[84] Beaunée G, Vergu E, Joly A, Ezanno P (2017). Controlling bovine paratuberculosis at a regional scale: towards a decision modeling tool. J Theor Biol.

[85] Viet A-F, Krebs S, Rat-Aspert O, Jeanpierre L, Belloc C, Ezanno P (2018). A modelling framework based on MDP to coordinate farmers’ disease control decisions at a regional scale. PLoS One.

[86] Wang T, Hennessy DA (2015). Strategic interactions among private and public efforts when preventing and stamping out a highly infectious animal disease. Am J Agri Econ.

[87] Tago D, Hammitt JK, Thomas A, Raboisson D (2016). The impact of farmers’ strategic behavior on the spread of animal infectious diseases. PLoS ONE.

[88] Probert WJM, Lakkur S, Fonnesbeck CJ, Shea K, Runge MC, Tildesley MJ, Ferrari MJ (2019). Context matters: using reinforcement learning to develop human-readable, state-dependent outbreak response policies. Phil Trans R Soc B.

[89] Liang R, Lu Y, Qu X, Su Q, Li C, Xia S, Liu Y, Zhang Q, Cao X, Chen Q, Niu B (2020). Prediction for global African swine fever outbreaks based on a combination of random forest algorithms and meteorological data. Transbound Emerg Dis.

[90] Salje H, Tran Kiem C, Lefrancq N, Courtejoie N, Bosetti P, Paireau J, Andronico A, Hozé N, Richet J, Dubost C-L, Le Strat Y, Lessler J, Levy Bruhl D, Fontanet A, Opatowski L, Boelle P-Y, Cauchemez S (2020). Estimating the burden of SARS-CoV-2 in France. Science.

[91] Parlavantzas N, Pham LM, Morin C, Arnoux S, Beaunée G, Qi L, Gontier P, Ezanno P (2019). A service-based framework for building and executing epidemic simulation applications in the cloud. Concurr Comp Pract Exper.

[92] Shah N, Malensek M, Shah H, Pallickara S, Pallickara SL (2019). Scalable network analytics for characterization of outbreak influence in voluminous epidemiology datasets. Concurr Comp Pract Exper.

[93] Han BA, Majumdar S, Calmon FP, Glicksberg BS, Horesh R, Kumar A, Perer A, von Marschall EB, Wei D, Mojsilović A, Varshney KR (2019). Confronting data sparsity to identify potential sources of Zika virus spillover infection among primates. Epidemics.

[94] Reddy S, Fox J, Purohit MP (2019). Artificial intelligence-enabled healthcare delivery. J R Soc Med.

[95] Duboz R, Echaubard P, Promburom P, Kilvington M, Ross H, Allen W, Ward J, Deffuant G, de Garine-Wichatitsky M, Binot A (2018). Systems thinking in practice: participatory modelling as a foundation for integrated approaches to health. Front Vet Sci.

[96] Van der Waal K, Morrison RB, Neuhauser C, Vilalta C, Perez AM (2017). Translating big data into smart data for veterinary epidemiology. Front Vet Sci.

[97] Reichman OJ, Jones MB, Schildhauer MP (2011). Challenges and opportunities of open data in ecology. Science.

[98] Schultze JL (2015). Teaching ‘big data’ analysis to young immunologists. Nat Immunol.

