# Supplementary material for: Research perspectives on animal health in the era of artificial intelligence
Source: Vet Res. 2021 Mar 6;52:40. doi: 10.1186/s13567-021-00902-4 (PMC7936489; doi:10.1186/s13567-021-00902-4)
Supplement: Supplementary file 1 — Additional file 1. Systematic literature review, interviews, previous publication in French. [file 13567_2021_902_MOESM1_ESM.doc]

**A - Literature review**

Publications were searched in PubMed and ISI Web of Knowledge (WoK) up to the 8^th^ of April 2020. Keywords were searched in titles, author keywords and publication summaries. Only articles in English have been kept. In ISI WoK, all databases were mobilised, but research categories that were outside the theme were removed to limit the number of abstracts to be screened. Duplicates were removed (same authors, title, year, support) when reading titles and abstracts.

Keywords searched: (health OR disease OR pathogen OR epidem*) AND (“artificial intelligence” OR “machine learning” OR “multi-agent” OR “multilevel agent” OR “markov decision process” OR “hidden markov”) AND (animal OR livestock OR cattle OR pig OR poultry OR wildlife) NOT cancer

Publications that were visibly off-topic when reading titles and abstracts were withdrawn, and then again when reading the articles. Other publications that did not appear on these databases but were known by the authors to be in this theme have been added. In the end, 197 publications were selected (Table and list thereafter).

**Table** Selection of articles published at the interface between artificial intelligence and animal health.

| **Databases** | **PubMed** | **Web of Knowledge** | **Complementary** | **Total** |
| --- | --- | --- | --- | --- |
| Number of identified articles | 182 | 406 |  | * |
| Number of selected articles (based on titles & abstracts) | 66 | 150 | 38 | 197 |

^*^There were duplicates between the two databases, so the total is not calculable before reading the titles and abstracts.

List of the 197 publications retained:

1. Abeysekera, S; Ooi, MPL; Kuang, YC; Tan, CP; Hassan, SS. 2014. Detecting Spongiosis in Stained Histopathological Specimen using Multispectral Imaging and Machine Learning. In: 9^th^ IEEE Sensors Applications Symposium (SAS), Queenstown, NEW ZEALAND
2. Adams, LG; Khare, S; Lawhon, SD; Rossetti, CA; Lewin, HA; Lipton, MS; Turse, JE; Wylie, DC; Bai, Y; Drake, KL. 2010. Enhancing the role of veterinary vaccines reducing zoonotic diseases of humans: Linking systems biology with vaccine development. In: Vaccine Technology III - An ECI Conference, Puerto Vallarta, MEXICO, doi:10.1016/j.vaccine.2011.05.080
3. Al-Rubaye, Z; Al-Sherbaz, A; McCormick, W; Turner, S. 2017. Sensor Data Classification for the Indication of Lameness in Sheep. In: Lecture Notes of the Institute for Computer Sciences Social Informatics and Telecommunications Engineering, 13^th^ European-Alliance-for-Innovation (EAI) International Conference on Collaborative Computing - Networking, Applications and Worksharing (CollaborateCom), Edinburgh, SCOTLAND, doi:10.1007/978-3-030-00916-8_29
4. Alsaaod, M; Romer, C; Kleinmanns, J; Hendriksen, K; Rose-Meierhofer, S; Plumer, L; Buscher, W. 2012. Electronic detection of lameness in dairy cows through measuring pedometric activity and lying behavior. APPLIED ANIMAL BEHAVIOUR SCIENCE, doi:10.1016/j.applanim.2012.10.001
5. Alvarez, JR; Arroqui, M; Mangudo, P; Toloza, J; Jatip, D; Rodriguez, JM; Teyseyre, A; Sanz, C; Zunino, A; Machado, C; Mateos, C. 2018. Body condition estimation on cows from depth images using Convolutional Neural Networks. COMPUTERS AND ELECTRONICS IN AGRICULTURE, doi:10.1016/j.compag.2018.09.039
6. Amouroux, E; Desvaux, S; Drogoul, A. 2008. Towards Virtual Epidemiology: An Agent-Based Approach to the Modeling of H5N1 Propagation and Persistence in North-Vietnam. In: Lecture Notes in Artificial Intelligence, 11^th^ Pacific Rim International Conference on Multi-Agents, Hanoi, VIETNAM.
7. Amrine, DE; White, BJ; Larson, RL. 2014. Comparison of classification algorithms to predict outcomes of feedlot cattle identified and treated for bovine respiratory disease. COMPUTERS AND ELECTRONICS IN AGRICULTURE, doi:10.1016/j.compag.2014.04.009
8. Anjara, F; Jaharadak, AA. 2018. Expert System for Diseases Diagnosis in Living Things: A Narrative Review. Journal of Physics Conference Series, 2nd Forum in Research, Science, and Technology, Palembang, INDONESIA, doi:10.1088/1742-6596/1167/1/012070
9. Anvar, SY; Tucker, A; Vinciotti, V; Venema, A; van Ommen, GJB; van der Maarel, SM; Raz, V; 't Hoen, PAC. 2011. Interspecies Translation of Disease Networks Increases Robustness and Predictive Accuracy. PLOS COMPUTATIONAL BIOLOGY e1002258, doi:10.1371/journal.pcbi.1002258
10. Arsevska E., Roche M., Hendrikx P., Chavernac D., Falala S., Lancelot R., Dufour B. 2016. Identification of terms for detecting early signals of emerging infectious disease outbreaks on the web. Comput. Electron. Agric., 123, 104-115, doi:10.1016/j.compag.2016.02.010
11. Arsevska E., Valentin S., Rabatel J., de Goër de Hervé J., Falala S., Lancelot R., Roche M. 2018. Web monitoring of emerging animal infectious diseases integrated in the French Animal Health Epidemic Intelligence System. PLoS One, 13 (8), doi:10.1371/journal.pone.0199960 (e0199960).
12. Awasthi, Amruta; Awasthi, Anshul; Riordan, Daniel; Walsh, Joseph. 2016. Non-Invasive Sensor Technology for the Development of a Dairy Cattle Health Monitoring System. COMPUTERS. 5, doi:10.3390/computers5040023.
13. Awaysheh A, Wilcke J, Elvinger F, Rees L, Fan W, Zimmerman K. 2018. Identifying free-text features to improve automated classification of structured histopathology reports for feline small intestinal disease. J Vet Diagn Invest. 30(2):211-217. doi: 10.1177/1040638717744002
14. Babayan, SA; Orton, RJ; Streicker, DG. 2018. Predicting reservoir hosts and arthropod vectors from evolutionary signatures in RNA virus genomes. SCIENCE, doi:10.1126/science.aap9072
15. Bailly X. 2017. Hidden markov phylogenetic models offer an interesting perspective to identify “high risk lineages” of environmental pathogens. Infection, Genetics and Evolution.
16. Bancroft, BA; Han, BA; Searle, CL; Biga, LM; Olson, DH; Kats, LB; Lawler, JJ; Blaustein, AR. 2011. Species-level correlates of susceptibility to the pathogenic amphibian fungus Batrachochytrium dendrobatidis in the United States. BIODIVERSITY AND CONSERVATION, doi:10.1007/s10531-011-0066-4
17. Beaunée G., Vergu E., Ezanno P. 2015. Modelling of paratuberculosis spread between dairy cattle farms at a regional scale. Veterinary Research 46:111. DOI: DOI 10.1186/s13567-015-0247-3.
18. Beaunée G., Vergu E., Joly A., Ezanno P. 2017. Controlling bovine paratuberculosis at a regional scale: towards a decision modeling tool. J. Theor. Biol. 435:157-183. doi: 10.1016/j.jtbi.2017.09.012.
19. Behmann, J; Hendriksen, K; Muller, U; Buscher, W; Plumer, L. 2016. Support Vector machine and duration-aware conditional random field for identification of spatio-temporal activity patterns by combined indoor positioning and heart rate sensors. GEOINFORMATICA, doi:10.1007/s10707-016-0260-3
20. Benitez, NG; Senti, VE; Tarke, AR. 2017. The diagnosis of the Fasciolosis bovine based on cast a net Bayesians. AVANCES 19, 1, 12-22
21. Benjamin, M; Yik, S. 2019. Precision Livestock Farming in Swine Welfare: A Review for Swine Practitioners ANIMALS, doi:10.3390/ani9040133
22. Bollig N, Clarke L, Elsmo E, Craven M. 2020. Machine learning for syndromic surveillance using veterinary necropsy reports. PLoS One 15(2):e0228105. doi: 10.1371/journal.pone.0228105.
23. Bonneau, M; Vayssade, JA; Troupe, W; Arquet, R. 2020. Outdoor animal tracking combining neural network and time-lapse cameras. COMPUTERS AND ELECTRONICS IN AGRICULTURE, doi:10.1016/j.compag.2019.105150
24. Bradley, R; Tagkopoulos, I; Kim, M; Kokkinos, Y; Panagiotakos, T; Kennedy, J; De Meyer, G; Watson, P; Elliott, J. 2019. Predicting early risk of chronic kidney disease in cats using routine clinical laboratory tests and machine learning. JOURNAL OF VETERINARY INTERNAL MEDICINE, doi:10.1111/jvim.15623
25. Bui T.M.A., Stinckwich S., Ziane M., Roche B., Ho T.V. 2015. KENDRICK: A Domain Specific Language and platform for mathematical epidemiological modelling. In: IEEE RIVF International Conference on Computing and Communication Technologies, Research, Innovation, and Vision for the Future. pp. 132–137. doi:10.1109/RIVF.2015.7049888
26. Busin, V; Viora, L; King, G; Tomlinson, M; LeKernec, J; Jonsson, N; Fioranelli, F. 2019. Evaluation of lameness detection using radar sensing in ruminants. VETERINARY RECORD, doi:10.1136/vetrec-2019-105407
27. Buyukyilmaz, M; Cibikdiken, AO; Abdalla, MAE; Seker, H. 2017. Identification of Chicken Eimeria Species from Microscopic Images by Using MLP Deep Learning Algorithm. In: International Conference on Video and Image Processing (ICVIP), SINGAPORE, doi:10.1145/3177404.3177445
28. Camanes G., Joly A., Fourichon C., Ben Romdhane R., Ezanno P. 2018. Control measures to avoid increase of paratuberculosis prevalence in dairy cattle herds: an individual-based modelling approach. Vet. Res. 49:60, doi:10.1186/s13567-018-0557-3.
29. Carroll, BT; Anderson, DV; Daley, W; Harbert, S; Britton, DF; Jackwood, MW. 2014. Detecting Symptoms of Diseases in Poultry through Audio Signal Processing. In: IEEE Global Conference on Signal and Information Processing (GlobalSIP), Atlanta, GA.
30. Casella, E; Khamesi, AR; Silvestri, S. 2019. Smartwatch Application for Horse Gaits Activity Recognition. In: 5^th^ IEEE International Conference on Smart Computing (SMARTCOMP), Washington, DC, doi:10.1109/SMARTCOMP.2019.00080
31. Cha, Kyung Jin. 2018. Animal Infectious Diseases Prevention through Big Data and Deep Learning. Journal of Intelligence and Information Systems, 24, 4, 137-154.
32. Chades, Iadine; Martin, Tara G.; Nicol, Samuel; Burgman, Mark A.; Possingham, Hugh P.; Buckley, Yvonne M. 2011. General rules for managing and surveying networks of pests, diseases, and endangered species. PROCEEDINGS OF THE NATIONAL ACADEMY OF SCIENCES OF THE UNITED STATES OF AMERICA, 108, 20, 8323-8328.
33. Charest, J; Beaudoin, JF; Cadorette, J; Lecomte, R; Brunet, CA; Fontaine, R. 2013. Preliminary Results of an Automatic Channel Fault Detection System on a Small Animal APD-Based Digital PET Scanner. In: 60^th^ IEEE Nuclear Science Symposium (NSS) / Medical Imaging Conference (MIC) / 20^th^ International Workshop on Room-Temperature Semiconductor X-ray and Gamma-ray Detectors, Seoul, SOUTH KOREA
34. Charest, J; Beaudoin, JF; Cadorette, J; Lecomte, R; Brunet, CA; Fontaine, R. 2014. Automatic Channel Fault Detection on a Small Animal APD-Based Digital PET Scanner. IEEE TRANSACTIONS ON NUCLEAR SCIENCE, doi:10.1109/TNS.2014.2346135
35. Charras-Garrido, M; Abrial, D; De Goer, J; Dachian, S; Peyrard, N. 2012. Classification method for disease risk mapping based on discrete hidden Markov random fields. BIOSTATISTICS, doi:10.1093/biostatistics/kxr043
36. Charras-Garrido, M; Azizi, L; Forbes, F; Doyle, S; Peyrard, N; Abrial, D. 2013. On the difficulty to delimit disease risk hot spots. INTERNATIONAL JOURNAL OF APPLIED EARTH OBSERVATION AND GEOINFORMATION, doi:10.1016/j.jag.2012.04.005
37. Chelotti, JO; Vanrell, SR; Galli, JR; Giovanini, LL; Rufiner, HL. 2018. A pattern recognition approach for detecting and classifying jaw movements in grazing cattle. COMPUTERS AND ELECTRONICS IN AGRICULTURE, doi:10.1016/j.compag.2017.12.013
38. Chen, J. 2013. Poultry Disease Data Mining Platform Based on Strategy Pattern. In: Communications in Computer and Information Science, 4^th^ International Conference on Information Computing and Applications (ICICA), Singapore
39. Chenar, S.S.; Deng, Z. 2018. Development of genetic programming-based model for predicting oyster norovirus outbreak risks. Water Res. 128, 20-37, doi:10.1016/j.watres.2017.10.032
40. Cheng, YH. 2019. A Development Architecture for the Intelligent Animal Care and Management System Based on the Internet of Things and Artificial Intelligence. In: 1st International Conference on Artificial Intelligence in Information and Communication (ICAIIC), Okinawa, JAPAN
41. Choquet, R; Carrie, C; Chambert, T; Boulinier, T. 2013. Estimating transitions between states using measurements with imperfect detection: application to serological data. ECOLOGY, doi:10.1890/12-1849.1
42. Chyba M, Coron JM, Mileyko Y, Rezaei H. 2016. Optimization of prion assemblies fragmentation. IEEE Decision and Control.
43. Coly S., Yao A.-F., Abrial D., Garrido M. 2016. Distributions to model overdispersed count data. Journal de la Société Française de Statistique, 157(2), 39-63.
44. Comin, A; Jeremiasson, A; Kratzer, G; Keeling, L. 2019. Revealing the structure of the associations between housing system, facilities, management and welfare of commercial laying hens using Additive Bayesian Networks. PREVENTIVE VETERINARY MEDICINE, doi:10.1016/j.prevetmed.2019.01.004
45. Conn, PB; Cooch, EG. 2009. Multistate capture-recapture analysis under imperfect state observation: an application to disease models. JOURNAL OF APPLIED ECOLOGY, doi:10.1111/j.1365-2664.2008.01597.x
46. Cowton, J; Kyriazakis, I; Bacardit, J. 2019. Automated Individual Pig Localisation, Tracking and Behaviour Metric Extraction Using Deep Learning. IEEE ACCESS, doi:10.1109/ACCESS.2019.2933060
47. Crispell, J; Benton, CH; Balaz, D; De Maio, N; Ahkmetova, A; Allen, A; Biek, R; Presho, EL; Dale, J; Hewinson, G; Lycett, SJ; Nunez-Garcia, J; Skuce, RA; Trewby, H; Wilson, DJ; Zadoks, RN; Delahay, RJ; Kao, RR. 2019. Combining genomics and epidemiology to analyse bi-directional transmission of Mycobacterium bovis in a multi-host system. ELIFE e45833, doi:10.7554/eLife.45833
48. Cuellar, AC; Kjaer, LJ; Baum, A; Stockmarr, A; Skovgard, H; Nielsen, SA; Andersson, MG; Lindstrom, A; Chirico, J; Luhken, R; Steinke, S; Kiel, E; Gethmann, J; Conraths, FJ; Larska, M; Smreczak, M; Orlowska, A; Hamnes, I; Sviland, S; Hopp, P; Brugger, K; Rubel, F; Balenghien, T; Garros, C; Rakotoarivony, I; Allene, X; Lhoir, J; Chavernac, D; Delecolle, JC; Mathieu, B; Delecolle, D; Setier-Rio, ML; Venail, R; Scheid, B; Chueca, MAM; Barcelo, C; Lucientes, J; Estrada, R; Mathis, A; Tack, W; Bodker, R. 2018. Monthly variation in the probability of presence of adult Culicoides populations in nine European countries and the implications for targeted surveillance. PARASITES & VECTORS, doi:10.1186/s13071-018-3182-0
49. Cui, Na; Chen, Yuguo; Small, Dylan S. 2013. Modeling Parasite Infection Dynamics when there Is Heterogeneity and Imperfect Detectability. BIOMETRICS, 69, 3, 683-692, doi:10.1111/biom.12050.
50. Dahlhausen, KE; Doroud, L; Firl, AJ; Polkinghorne, A; Eisen, JA. 2018. Characterization of shifts of koala (Phascolarctos cinereus) intestinal microbial communities associated with antibiotic treatment. PEERJ e4452, doi:10.7717/peerj.4452
51. de Almeida, Sandro Jeronimo; Martins Ferreira, Ricardo Poley; Eiras, Alvaro E.; Obermayr, Robin P.; Geier, Martin. 2010. Multi-agent modeling and simulation of an Aedes aegypti mosquito population. ENVIRONMENTAL MODELLING & SOFTWARE, 25, 12, 1490-1507, doi:10.1016/j.envsoft.2010.04.021.
52. Detilleux, JC. 2008. The analysis of disease biomarker data using a mixed hidden Markov model. GENETICS SELECTION EVOLUTION, doi:10.1051/gse:2008017
53. Dhoble, AS; Ryan, KT; Lahiri, P; Chen, M; Pang, XX; Cardoso, FC; Bhalerao, KD. 2019. Cytometric fingerprinting and machine learning (CFML): A novel label-free, objective method for routine mastitis screening. COMPUTERS AND ELECTRONICS IN AGRICULTURE, doi:10.1016/j.compag.2019.04.029
54. Dion, E; Lambin, EF. 2012. Scenarios of transmission risk of foot-and-mouth with climatic, social and landscape changes in southern Africa. APPLIED GEOGRAPHY, doi:10.1016/j.apgeog.2012.05.001
55. Dion, E; VanSchalkwyk, L; Lambin, EF. 2011. The landscape epidemiology of foot-and-mouth disease in South Africa: A spatially explicit multi-agent simulation. ECOLOGICAL MODELLING, doi:10.1016/j.ecolmodel.2011.03.026
56. Domun, Y; Pedersen, LJ; White, D; Adeyemi, O; Norton, T. 2019. Learning patterns from time-series data to discriminate predictions of tail-biting, fouling and diarrhoea in pigs. COMPUTERS AND ELECTRONICS IN AGRICULTURE, doi:10.1016/j.compag.2019.104878
57. Dorea, FC; Muckle, CA; Kelton, D; McClure, JT; McEwen, BJ; McNab, WB; Sanchez, J; Revie, CW. 2013. Exploratory Analysis of Methods for Automated Classification of Laboratory Test Orders into Syndromic Groups in Veterinary Medicine. PLOS ONE e57334, doi :10.1371/journal.pone.0057334
58. Dzobo K, Adotey S, Thomford NE, Dzobo W. 2019. Integrating Artificial and Human Intelligence: A Partnership for Responsible Innovation in Biomedical Engineering and Medicine. OMICS, doi: 10.1089/omi.2019.0038.
59. Ebrahimie, E; Ebrahimi, F; Ebrahimi, M; Tomlinson, S; Petrovski, KR. 2018. A large-scale study of indicators of sub-clinical mastitis in dairy cattle by attribute weighting analysis of milk composition features: highlighting the predictive power of lactose and electrical conductivity. JOURNAL OF DAIRY RESEARCH, doi:10.1017/S0022029918000249
60. Ebrahimie, Esmaeil; Ebrahimi, Faezeh; Ebrahimi, Mansour; Tomlinson, Sarah; Petrovski, Kiro R. 2018. Hierarchical pattern recognition in milking parameters predicts mastitis prevalence. COMPUTERS AND ELECTRONICS IN AGRICULTURE 147, 6, 11, doi:10.1016/j.compag.2018.02.003
61. Ehret, A; Hochstuhl, D; Krattenmacher, N; Tetens, J; Klein, MS; Gronwald, W; Thaller, G. 2015. Short communication: Use of genomic and metabolic information as well as milk performance records for prediction of subclinical ketosis risk via artificial neural networks. JOURNAL OF DAIRY SCIENCE, doi:10.3168/jds.2014-8602
62. Eng, CLP; Tong, JC; Tan, TW. 2014. Predicting host tropism of influenza A virus proteins using random forest. BMC MEDICAL GENOMICS, doi:10.1186/1755-8794-7-S3-S1
63. Eng, CLP; Tong, JC; Tan, TW. 2016. Distinct Host Tropism Protein Signatures to Identify Possible Zoonotic Influenza A Viruses. PLOS ONE e0150173, doi:10.1371/journal.pone.0150173
64. Eng, CLP; Tong, JC; Tan, TW. 2017. Predicting Zoonotic Risk of Influenza A Viruses from Host Tropism Protein Signature Using Random Forest. INTERNATIONAL JOURNAL OF MOLECULAR SCIENCES, doi:10.3390/ijms18061135
65. Esener N., Green M.J., Emes R.D., Jowett B., Davies P.L., Bradley A.J., Dottorini T. 2018. Discrimination of contagious and environmental strains of Streptococcus uberis in dairy herds by means of mass spectrometry and machine-learning. Scientific Report 8:17517, doi:10.1038/s41598-018-35867-6
66. Ferris, MC; Christensen, A; Wangen, SR. 2020. Symposium review: Dairy Brain-Informing decisions on dairy farms using data analytics. JOURNAL OF DAIRY SCIENCE, doi:10.3168/jds.2019-17199
67. Forbes, F; Charras-Garrido, M; Azizi, L; Doyle, S; Abrial, D. 2013. SPATIAL RISK MAPPING FOR RARE DISEASE WITH HIDDEN MARKOV FIELDS AND VARIATIONAL EM. ANNALS OF APPLIED STATISTICS, doi:10.1214/13-AOAS629
68. Fountain-Jones, NM; Craft, ME; Funk, WC; Kozakiewicz, C; Trumbo, DR; Boydston, EE; Lyren, LM; Crooks, K; Lee, JS; VandeWoude, S; Carver, S. 2017. Urban landscapes can change virus gene flow and evolution in a fragmentation-sensitive carnivore. MOLECULAR ECOLOGY, doi:10.1111/mec.14375
69. Fountain-Jones, NM; Machado, G; Carver, S; Packer, C; Recamonde-Mendoza, M; Craft, ME. 2019. How to make more from exposure data? An integrated machine learning pipeline to predict pathogen exposure. JOURNAL OF ANIMAL ECOLOGY, doi:10.1111/1365-2656.13076
70. Franzo G, Corso B, Tucciarone CM, Drigo M, Caldin M, Cecchinato M. 2020. Comparison and validation of different models and variable selection methods for predicting survival after canine parvovirus infection. Vet Rec. doi: 10.1136/vr.105283.
71. Frohlich, H; Claes, K; De Wolf, C; Van Damme, X; Michel, A. 2018. A Machine Learning Approach to Automated Gait Analysis for the Noldus Catwalk System. IEEE TRANSACTIONS ON BIOMEDICAL ENGINEERING, doi:10.1109/TBME.2017.2701204
72. Ganz, HH; Doroud, L; Firl, AJ; Hird, SM; Eisen, JA; Boyce, WM. 2017. Community-Level Differences in the Microbiome of Healthy Wild Mallards and Those Infected by Influenza A Viruses. MSYSTEMS e00188-16, doi:10.1128/mSystems.00188-16
73. Gattani, A; Singh, SV; Agrawal, A; Khan, MH; Singh, P. 2019. Recent progress in electrochemical biosensors as point of care diagnostics in livestock health. ANALYTICAL BIOCHEMISTRY, doi:10.1016/j.ab.2019.05.014
74. Gautam, A; Sharma, A; Jaiswal, S; Fatma, S; Arora, V; Iquebal, MA; Nandi, S; Sundaray, JK; Jayasankar, P; Rai, A; Kumar, D. 2016. Development of Antimicrobial Peptide Prediction Tool for Aquaculture Industries. PROBIOTICS AND ANTIMICROBIAL PROTEINS, doi:10.1007/s12602-016-9215-0
75. Giesert, AL; Balke, WT; Jahns, G. 2011. Probabilistic analysis of coughs in pigs to diagnose respiratory infections. LANDBAUFORSCHUNG 61, 3, 237-242
76. Golden, CE; Rothrock, MJ; Mishra, A. 2019. Using Farm Practice Variables as Predictors of Listeria spp. Prevalence in Pastured Poultry Farms. FRONTIERS IN SUSTAINABLE FOOD SYSTEMS, doi:10.3389/fsufs.2019.00015
77. Gong, HS; Weon, S; Huh, JH. 2018. A Study on the Design of Humane Animal Care System and Java Implementation. JOURNAL OF INFORMATION PROCESSING SYSTEMS, doi:10.3745/JIPS.02.0096
78. Gonzalez-Recio, O; Forni, S. 2011. Genome-wide prediction of discrete traits using bayesian regressions and machine learning. GENETICS SELECTION EVOLUTION, doi:10.1186/1297-9686-43-7
79. Goodswen, SJ; Kennedy, PJ; Ellis, JT. 2017. On the application of reverse vaccinology to parasitic diseases: a perspective on feature selection and ranking of vaccine candidates. INTERNATIONAL JOURNAL FOR PARASITOLOGY, doi:10.1016/j.ijpara.2017.08.004
80. Gorczyca, MT; Gebremedhin, KG. 2020. Ranking of environmental heat stressors for dairy cows using machine learning algorithms. COMPUTERS AND ELECTRONICS IN AGRICULTURE JAN, doi:10.1016/j.compag.2019.105124
81. Gupta, RK; Ruhil, AP; Lathwal, SS; Mohanty, TK; Singh, Y. 2014. Detection of Lameness of Cow based on Body Weight using Artificial Neural Network. In: 8^th^ International Conference on Computing for Sustainable Global Development (INDIACom), New Delhi, INDIA
82. Guy, R; Laredo, C; Vergu, E. 2015. Approximation of epidemic models by diffusion processes and their statistical inference. JOURNAL OF MATHEMATICAL BIOLOGY, 70, 3, 621-646, doi:10.1007/s00285-014-0777-8
83. Han, BA; Majumdar, S; Calmon, FP; Glicksberg, BS; Horesh, R; Kumar, A; Perer, A; von Marschall, EB; Wei, D; Mojsilovic, A; Varshney, KR. 2019. Confronting data sparsity to identify potential sources of Zika virus spillover infection among primates. EPIDEMICS, doi:10.1016/j.epidem.2019.01.005
84. Han, BA; Schmidt, JP; Alexander, LW; Bowden, SE; Hayman, DTS; Drake, JM. 2016. Undiscovered Bat Hosts of Filoviruses. PLOS NEGLECTED TROPICAL DISEASES, doi:10.1371/journal.pntd.0004815
85. Hedell, R; Andersson, MG; Faverjon, C; Marcillaud-Pitel, C; Leblond, A; Mostad, P. 2019. Surveillance of animal diseases through implementation of a Bayesian spatio-temporal model: A simulation example with neurological syndromes in horses and West Nile Virus. PREVENTIVE VETERINARY MEDICINE, doi:10.1016/j.prevetmed.2018.11.010
86. Heidrich A, Schmidt J, Zimmermann J, Saluz HP. 2013. Automated Segmentation and Object Classification of CT Images: Application to In Vivo Molecular Imaging of Avian Embryos. Int J Biomed Imaging. doi: 10.1155/2013/508474.
87. Hepworth, PJ; Nefedov, AV; Muchnik, IB; Morgan, KL. 2012. Broiler chickens can benefit from machine learning: support vector machine analysis of observational epidemiological data. JOURNAL OF THE ROYAL SOCIETY INTERFACE, doi:10.1098/rsif.2011.0852
88. Hermans, K; Waegeman, W; Opsomer, G; Van Ranst, B; De Koster, J; Van Eetvelde, M; Hostens, M. 2017. Novel approaches to assess the quality of fertility data stored in dairy herd management software. JOURNAL OF DAIRY SCIENCE, doi:10.3168/jds.2016-11896
89. Herrick, KA; Huettmann, F; Lindgren, MA. 2013. A global model of avian influenza prediction in wild birds: the importance of northern regions. VETERINARY RESEARCH, doi:10.1186/1297-9716-44-42
90. Hollings, T; Robinson, A; van Andel, M; Jewell, C; Burgman, M. 2017. Species distribution models: A comparison of statistical approaches for livestock and disease epidemics. PLOS ONE e0183626, doi:10.1371/journal.pone.0183626
91. Holt, AC; Salkeld, DJ; Fritz, CL; Tucker, JR; Gong, P. 2009. Spatial analysis of plague in California: niche modeling predictions of the current distribution and potential response to climate change. INTERNATIONAL JOURNAL OF HEALTH GEOGRAPHICS, doi:10.1186/1476-072X-8-38
92. Hoscheit P., Geeraert S., Beaunée G., Monod H., Gilligan C.A.G, Filipe J., Vergu E., Moslonka-Lefebvre M. 2016. Dynamical Network Models for Cattle Trade: Towards Economy-Based Epidemic Risk Assessment. J. Complex Networks, cnw026, doi:10.1093/comnet/cnw026
93. Hur B, Hardefeldt LY, Verspoor K, Baldwin T, Gilkerson JR. 2019. Using natural language processing and VetCompass to understand antimicrobial usage patterns in Australia. Aust Vet J. doi: 10.1111/avj.12836
94. Hyde RM, Down PM, Bradley AJ, Breen JE, Hudson C, Leach KA, Green MJ. 2020. Automated prediction of mastitis infection patterns in dairy herds using machine learning. Sci Rep. 10(1):4289. doi: 10.1038/s41598-020-61126-8.
95. Iwasaki, W; Ishida, S; Kondo, D; Ito, Y; Tateno, J; Tomioka, M. 2019. Monitoring of the core body temperature of cows using implantable wireless thermometers. COMPUTERS AND ELECTRONICS IN AGRICULTURE, doi:10.1016/j.compag.2019.06.004
96. Jahns, G. 2008. Call recognition to identify cow conditions - A call-recogniser translating calls to text. COMPUTERS AND ELECTRONICS IN AGRICULTURE, doi:10.1016/j.compag.2007.09.005
97. Jensen, DB; Larsen, MLV; Pedersen, LJ. 2020. Predicting pen fouling in fattening pigs from pig position. LIVESTOCK SCIENCE, doi:10.1016/j.livsci.2019.103852
98. Jones-Diette, JS; Dean, RS; Cobb, M; Brennan, ML. 2019. Validation of text-mining and content analysis techniques using data collected from veterinary practice management software systems in the UK. PREVENTIVE VETERINARY MEDICINE, doi:10.1016/j.prevetmed.2019.02.015
99. Kaler, J; Mitsch, J; Vazquez-Diosdado, JA; Bollard, N; Dottorini, T; Ellis, KA. 2020. Automated detection of lameness in sheep using machine learning approaches: novel insights into behavioural differences among lame and non-lame sheep. ROYAL SOCIETY OPEN SCIENCE, doi:10.1098/rsos.190824
100. Kargarfard, F; Sami, A; Mohammadi-Dehcheshmeh, M; Ebrahimie, E. 2016. Novel approach for identification of influenza virus host range and zoonotic transmissible sequences by determination of host-related associative positions in viral genome segments. BMC GENOMICS, doi:10.1186/s12864-016-3250-9
101. Keceli, AS; Catal, C; Kaya, A; Tekinerdogan, B. 2020. Development of a recurrent neural networks-based calving prediction model using activity and behavioral data. COMPUTERS AND ELECTRONICS IN AGRICULTURE, doi:10.1016/j.compag.2020.105285
102. Kerkow, A; Wieland, R; Fruh, L; Holker, F; Jeschke, JM; Werner, D; Kampen, H. 2020. Can data from native mosquitoes support determining invasive species habitats? Modelling the climatic niche of Aedes japonicus japonicus (Diptera, Culicidae) in Germany. PARASITOLOGY RESEARCH, doi:10.1007/s00436-019-06513-5
103. Kerkow, A; Wieland, R; Koban, MB; Holker, F; Jeschke, JM; Werner, D; Kampen, H. 2019. What makes the Asian bush mosquito Aedes japonicus japonicus feel comfortable in Germany? A fuzzy modelling approach. PARASITES & VECTORS, doi:10.1186/s13071-019-3368-0
104. Khalil, H; Olsson, G; Magnusson, M; Evander, M; Hornfeldt, B; Ecke, F. 2017. Spatial prediction and validation of zoonotic hazard through micro-habitat properties: where does Puumala hantavirus hole - up? BMC INFECTIOUS DISEASES, doi:10.1186/s12879-017-2618-z
105. Kim, T; Hwang, W; Zhang, AD; Sen, S; Ramanathan, M. 2010. Multi-agent modeling of the South Korean avian influenza epidemic. BMC INFECTIOUS DISEASES, doi:10.1186/1471-2334-10-236
106. Knowler, SP; Dumas, E; Spiteri, M; McFadyen, AK; Stringer, F; Wells, K; Rusbridge, C. 2020. Facial changes related to brachycephaly in Cavalier King Charles Spaniels with Chiari-like malformation associated pain and secondary syringomyelia. JOURNAL OF VETERINARY INTERNAL MEDICINE, doi:10.1111/jvim.15632
107. Koons, DN; Gamelon, M; Gaillard, JM; Aubry, LM; Rockwell, RF; Klein, F; Choquet, R; Gimenez, O. 2014. Methods for studying cause-specific senescence in the wild. METHODS IN ECOLOGY AND EVOLUTION, doi:10.1111/2041-210X.12239
108. Kuncheva, LI; Vilas, VJD; Rodriguez, JJ. 2007. Diagnosing scrapie in sheep: A classification experiment. COMPUTERS IN BIOLOGY AND MEDICINE, doi:10.1016/j.compbiomed.2006.10.011
109. Laperriere, V; Brugger, K; Rubel, F. 2016. Cross-scale modeling of a vector-borne disease, from the individual to the metapopulation: The seasonal dynamics of sylvatic plague in Kazakhstan. ECOLOGICAL MODELLING, 342, 34-48, doi:10.1016/j.ecolmodel.2016.09.023
110. Lewis, FI; Brulisauer, F; Gunn, GJ. 2011. Structure discovery in Bayesian networks: An analytical tool for analysing complex animal health data. PREVENTIVE VETERINARY MEDICINE, doi:10.1016/j.prevetmed.2011.02.003
111. Li, L; Gao, Q; Mao, X; Cao, Y. 2014. New support vector machine-based method for microRNA target prediction. GENETICS AND MOLECULAR RESEARCH, doi:10.4238/2014.June.9.3
112. Liakos, KG; Busato, P; Moshou, D; Pearson, S; Bochtis, D. 2018. Machine Learning in Agriculture: A Review. SENSORS, doi:10.3390/s18082674
113. Liang, R.; Lu, Y.; Qu, X.; Su, Q.; Li, C.; Xia, S.; Liu, Y.; Zhang, Q.; Cao, X.; Chen, Q., et al. 2020. Prediction for global African swine fever outbreaks based on a combination of random forest algorithms and meteorological data. Transboundary Emer. Dis. 67, 935-946, doi:10.1111/tbed.13424
114. Lopez-Cortes, XA.; Nachtigall, FM.; Olate, VR.; Araya, M; Oyanedel, S; Diaz, V; Jakob, E; Rios-Momberg, M; Santos, LS. 2017. Fast detection of pathogens in salmon farming industry. AQUACULTURE. 470. 17. 24, doi:10.1016/j.aquaculture.2016.12.008
115. Lupolova, N; Dallman, TJ; Holden, NJ; Gally, DL. 2017. Patchy promiscuity: machine learning applied to predict the host specificity of Salmonella enterica and Escherichia coli. MICROBIAL GENOMICS, doi:10.1099/mgen.0.000135
116. Lupolova, N; Dallman, TJ; Matthews, L; Bono, JL; Gally, DL. 2016. Support vector machine applied to predict the zoonotic potential of E-coli O157 cattle isolates. PROCEEDINGS OF THE NATIONAL ACADEMY OF SCIENCES OF THE UNITED STATES OF AMERICA, doi:10.1073/pnas.1606567113
117. Machado, G; Mendoza, MR; Corbellini, LG. 2015. What variables are important in predicting bovine viral diarrhea virus? A random forest approach. VETERINARY RESEARCH, doi:10.1186/s13567-015-0219-7
118. Machado, G; Vilalta, C; Recamonde-Mendoza, M; Corzo, C; Torremorell, M; Perez, A; VanderWaal, K. 2019. Identifying outbreaks of Porcine Epidemic Diarrhea virus through animal movements and spatial neighborhoods. SCIENTIFIC REPORTS, doi:10.1038/s41598-018-36934-8
119. Maclachlan, MJ; Springborn, MR; Fackler, PL. 2017. LEARNING ABOUT A MOVING TARGET IN RESOURCE MANAGEMENT: OPTIMAL BAYESIAN DISEASE CONTROL. AMERICAN JOURNAL OF AGRICULTURAL ECONOMICS, doi:10.1093/ajae/aaw033
120. Malensek, M; Budgaga, W; Stern, R; Pallickara, S; Pallickara, SL. 2019. Trident: Distributed Storage, Analysis, and Exploration of Multidimensional Phenomena. IEEE TRANSACTIONS ON BIG DATA, doi:10.1109/TBDATA.2018.2817505
121. Mammadova, NM.; Keskin, I. 2015. Application of neural network and adaptive neuro-fuzzy inference system to predict subclinical mastitis in dairy cattle. INDIAN JOURNAL OF ANIMAL RESEARCH. 49. 671. 679, doi:10.18805/ijar.5581
122. Mancia, A; Ryan, JC; Chapman, RW; Wu, QZ; Warr, GW; Gulland, FMD; Van Dolah, FM. 2012. Health status, infection and disease in California sea lions (Zalophus californianus) studied using a canine microarray platform and machine-learning approaches. DEVELOPMENTAL AND COMPARATIVE IMMUNOLOGY, doi:10.1016/j.dci.2011.10.011
123. Mancia, A; Ryan, JC; Van Dolah, FM; Kucklick, JR; Rowles, TK; Wells, RS; Rosel, PE; Hohn, AA; Schwacke, LH. 2014. Machine learning approaches to investigate the impact of PCBs on the transcriptome of the common bottlenose dolphin (Tursiops truncatus). MARINE ENVIRONMENTAL RESEARCH, doi:10.1016/j.marenvres.2014.03.007
124. Marsilio, S; Newman, SJ; Estep, JS; Giaretta, PR; Lidbury, JA; Warry, E; Flory, A; Morley, PS; Smoot, K; Seeley, EH; Powell, MJ; Suchodolski, JS; Steiner, JM. 2020. Differentiation of lymphocytic-plasmacytic enteropathy and small cell lymphoma in cats using histology-guided mass spectrometry. JOURNAL OF VETERINARY INTERNAL MEDICINE, doi:10.1111/jvim.15742
125. Miekley, B.; Traulsen, I.; Krieter, J. 2013. Mastitis detection in dairy cows: the application of support vector machines. JOURNAL OF AGRICULTURAL SCIENCE. 151. 889. 897, doi:10.1017/S0021859613000178
126. Milosevic, B; Ciric, S; Lalic, N; Milanovic, V; Savic, Z; Omerovic, I; Doskovic, V; Djordjevic, S; Andjusic, L. 2019. Machine learning application in growth and health prediction of broiler chickens. WORLDS POULTRY SCIENCE JOURNAL, doi:10.1017/S0043933919000254
127. Morales, IR; Cebrian, DR; Blanco, EF; Sierra, AP. 2016. Early warning in egg production curves from commercial hens: A SVM approach. COMPUTERS AND ELECTRONICS IN AGRICULTURE, doi:10.1016/j.compag.2015.12.009
128. Morota, G; Ventura, RV; Silva, FF; Koyama, M; Fernando, SC. 2018. BIG DATA ANALYTICS AND PRECISION ANIMAL AGRICULTURE SYMPOSIUM: Machine learning and data mining advance predictive big data analysis in precision animal agriculture. JOURNAL OF ANIMAL SCIENCE, doi:10.1093/jas/sky014
129. Moslonka-Lefebvre M, Gilligan CA, Monod H, Belloc C, Ezanno P, Filipe JAN, Vergu E. 2016. Market analyses of livestock trade networks to inform the prevention of joint economic and epidemiological risks. Journal of the Royal Society Interface.
130. Munoz, S; Guerrero, FD; Kellogg, A; Heekin, AM; Leung, MY. 2017. Bioinformatic prediction of G protein-coupled receptor encoding sequences from the transcriptome of the foreleg, including the Haller's organ, of the cattle tick, Rhipicephalus australis. PLOS ONE e0172326, doi:10.1371/journal.pone.0172326
131. Naghani, SY; Dara, R; Poljak, Z; Sharif, S. 2019. A review of knowledge discovery process in control and mitigation of avian influenza. ANIMAL HEALTH RESEARCH REVIEWS, doi:10.1017/S1466252319000033
132. Naranjo-Lucena, A; Corbalan, MPM; Martinez-Ibeas, AM; McGrath, G; Murray, G; Casey, M; Good, B; Sayers, R; Mulcahy, G; Zintl, A. 2018. Spatial patterns of Fasciola hepatica and Calicophoron daubneyi infections in ruminants in Ireland and modelling of C-daubneyi infection. PARASITES & VECTORS, doi:10.1186/s13071-018-3114-z
133. O’Hare A., Lycett S.J., Doherty T.M., Salvador L.C., Kao R.R. 2016. Broadwick: a framework for computational epidemiology. BMC Bioinformatics 17. doi:10.1186/s12859-016-0903-2
134. O'Mahony, N; Campbell, S; Carvalho, A; Krpalkova, L; Riordan, D; Walsh, J. 2019. 3D Vision for Precision Dairy Farming. In: 6^th^ International-Federation-of-Automatic-Control (IFAC) Conference on Sensing, Control and Automation Technologies for Agriculture (AGRICONTROL), Sydney, AUSTRALIA, doi:10.1016/j.ifacol.2019.12.555
135. Pandit P., Hoch T., Ezanno P., Beaudeau F., Vergu E. 2016. Q fever spread between dairy cattle herds in an enzootic region: modelling contributions of airborne transmission and trade. Vet. Res. 47:48, DOI 10.1186/s13567-016-0330-4.
136. Parez-Enciso M, Zingaretti LM. 2019. A Guide for Using Deep Learning for Complex Trait Genomic Prediction. Genes 10(7). pii: E553. doi: 10.3390/genes10070553.
137. Pastell, M; Frondelius, L. 2018. A hidden Markov model to estimate the time dairy cows spend in feeder based on indoor positioning data. COMPUTERS AND ELECTRONICS IN AGRICULTURE, doi:10.1016/j.compag.2018.07.005
138. Paul, PNT; Bah, A; Ndiaye, PI; Ndione, JA. 2018. An Agent Based Model for Studying the Impact of Rainfall on Rift Valley Fever Transmission at Ferlo (Senegal). In: Lecture Notes in Computer Science, 18^th^ International Conference on Computational Science and Its Applications (ICCSA), Melbourne, AUSTRALIA, doi:10.1007/978-3-319-95165-2_20
139. Pegorini V, Karam LZ, Pitta CS, Cardoso R, da Silva JC, Kalinowski HJ, Ribeiro R, Bertotti FL, Assmann TS. 2015. In Vivo Pattern Classification of Ingestive Behavior in Ruminants Using FBG Sensors and Machine Learning. Sensors 15(11):28456-71. doi: 10.3390/s151128456.
140. Perez, AM; Zeng, D; Tseng, CJ; Chen, HC; Whedbee, Z; Paton, D; Thurmond, MC. 2009. A web-based system for near real-time surveillance and space-time cluster analysis of foot-and-mouth disease and other animal diseases. PREVENTIVE VETERINARY MEDICINE, doi:10.1016/j.prevetmed.2009.05.006
141. Pfeiffer, DU; Stevens, KB. 2015. Spatial and temporal epidemiological analysis in the Big Data era. PREVENTIVE VETERINARY MEDICINE, doi:10.1016/j.prevetmed.2015.05.012
142. Pham L.M., Parlavantzas N., Morin C., Arnoux S., Qi L., Gontier P., Ezanno P. 2017. DiFFuSE, a distributed framework for cloud-based epidemic simulations: a case study in modelling the spread of bovine viral diarrhea virus. In: 9^th^ IEEE International Conference on Cloud Computing Technology and Science (CloudCom), Hong Kong, China, doi:10.1109/CloudCom.2017.41
143. Phan, MVT; Tri, TN; Anh, PH; Baker, S; Kellam, P; Cotten, M; Kiet, BT; Berto, A; Boni, MF; Bryant, JE; Phu, BD; Campbell, JI; Carrique-Mas, J; Hung, DM; Huong, DT; Oanh, DT; Day, JN; Tan, DV; Van Doorn, HR; Han, DA; Farrar, JJ; Trang, HTT; Nghia, HDT; Long, HB; Duong, HV; Thu, HTK; Cuong, LC; Hung, LM; Phuong, LT; Phuc, LT; Phuong, LT; Luat, LX; Ha, LTT; Chuong, LV; Loan, MTP; Nadjm, B; Bao, NT; Hoa, NT; Tu, NC; Thuan, ND; Dong, N; Chuyen, NK; An, NN; Vinh, NN; Hung, NQ; Dung, NT; Minh, NT; Binh, NT; Tham, NTH; Tien, NTH; Chuc, NTK; Ngoc, NTL; Ha, NTL; Lien, NTN; Diep, NTN; Nhung, NT; Chau, NTS; Chi, NTY; Trinh, NT; Van, NT; Van Cuong, N; Van Hung, N; Van Kinh, N; Hoang, NVM; Vanmy, N; Thang, NV; Thanh, NV; Chau, NVV; Xang, NV; My, PH; Khoa, PTM; Tam, PTT; Lao, PV; Minh, PV; Bay, PVB; Rabaa, MA; Rahman, M; Thompson, C; Thwaites, G; Ngan, TTD; Nhu, TDH; Chau, THM; Toan, TK; Phuc, TM; Hong, TTK; Dung, TTN; Thanh, TTT; Minh, TTT; Nguyen, TT; Hien, TT; Tri, TQ; Hien, VB; Tai, VN; Cuong, VQ; Phat, VV; Huong, VTL; Hang, VTT; Wertheim, H; Bogaardt, C; Chase-Topping, M; Ivens, A; Lu, L; Nyugen, D; Rambaut, A; Simmonds, P; Woolhouse, M; Munnink, BO; Deijs, M; van der Hoek, L; Jebbink, MF; Farsani, SMJ; Dodd, K; Euren, J; Lucas, A; Ortiz, N; Pennacchio, L; Rubin, E; Saylors, KE; Hai, TM; Wolfe, ND . 2018. Identification and characterization of Coronaviridae genomes from Vietnamese bats and rats based on conserved protein domains. VIRUS EVOLUTION, doi:10.1093/ve/vey035
144. Picault S, Huang YL, Sicard V, Ezanno P. 2017. Enhancing Sustainability of Epidemiological Models through a Generic Multilevel Agent-based Approach. In: IJCAI
145. Picault, S; Huang, YL; Sicard, V; Arnoux, S; Beaunee, G; Ezanno, P. 2019. EMULSION: Transparent and flexible multiscale stochastic models in human, animal and plant epidemiology. PLOS COMPUTATIONAL BIOLOGY e1007342, doi:10.1371/journal.pcbi.1007342
146. Picault, S; Huang, YL; Sicard, V; Beaudeau, F; Ezanno, P. 2017. A Multi-Level Multi-Agent Simulation Framework in Animal Epidemiology. In: Lecture Notes in Artificial Intelligence, 15^th^ International Conference on Practical Applications of Agents and Multi-Agent Systems (PAAMS), Porto, PORTUGAL, doi:10.1007/978-3-319-59930-4_17
147. Probert, WJM; Lakkur, S; Fonnesbeck, CJ; Shea, K; Runge, MC; Tildesley, MJ; Ferrari, MJ. 2019. Context matters: using reinforcement learning to develop human-readable, state-dependent outbreak response policies. PHILOSOPHICAL TRANSACTIONS OF THE ROYAL SOCIETY B-BIOLOGICAL SCIENCES, doi:10.1098/rstb.2018.0277
148. Qi L., Beaunée G., Arnoux S., Dutta B.L., Joly A., Vergu E., Ezanno P. 2019. Neighbourhood contacts and trade movements drive the regional spread of bovine viral diarrhoea virus (BVDV). Vet Res, 50:30, doi:10.1186/s13567-019-0647-x.
149. Rabatel, J; Arsevska, E; Roche, M. 2019. PADI-web corpus: Labeled textual data in animal health domain. DATA IN BRIEF, doi:10.1016/j.dib.2018.12.063
150. Ramaprasad, A; Mourier, T; Naeem, R; Malas, TB; Moussa, E; Panigrahi, A; Vermont, SJ; Otto, TD; Wastling, J; Pain, A. 2015. Comprehensive Evaluation of Toxoplasma gondii VEG and Neospora caninum LIV Genomes with Tachyzoite Stage Transcriptome and Proteome Defines Novel Transcript Features. PLOS ONE e0124473, doi:10.1371/journal.pone.0124473
151. Reulke, R; Ruess, D; Deckers, N; Barnewitz, D; Wieckert, A; Kienapfel, K. 2018. Analysis of Motion Patterns for Pain Estimation of Horses. In: 15^th^ IEEE International Conference on Advanced Video and Signal Based Surveillance (AVSS), Auckland, NEW ZEALAND
152. Robertson, C; Sawford, K; Gunawardana, WSN; Nelson, TA; Nathoo, F; Stephen, C. 2011. A Hidden Markov Model for Analysis of Frontline Veterinary Data for Emerging Zoonotic Disease Surveillance. PLOS ONE e24833, doi:10.1371/journal.pone.0024833
153. Roche, B; Guegan, JF; Bousquet, F. 2008. Multi-agent systems in epidemiology: a first step for computational biology in the study of vector-borne disease transmission. BMC BIOINFORMATICS, doi:10.1186/1471-2105-9-435
154. Rodriguez, Sara V.; Jensen, Tina Birk; Pla, Lluis M.; Kristensen, Anders Ringgaard. 2010. Optimal replacement policies and economic value of clinical observations in sow herds. LIVESTOCK SCIENCE, 138, 207-219, doi:10.1016/j.livsci.2010.12.026.
155. Romero MP, Chang YM, Brunton LA, Parry J, Prosser A, Upton P, Rees E, Tearne O, Arnold M, Stevens K, Drewe JA. 2019. Decision tree machine learning applied to bovine tuberculosis risk factors to aid disease control decision making. Prev Vet Med. 175:104860. doi: 10.1016/j.prevetmed.2019.104860.
156. Ruhnke, I; Boshoff, J; Cristiani, IV; Schneider, D; Welch, M; Sibanda, TZ; Kolakshyapati, M. 2019. Free-range laying hens: using technology to show the dynamics and impact of hen movement. ANIMAL PRODUCTION SCIENCE, doi:10.1071/AN19256
157. Sabbadin R, Viet AF. 2016. Leader-follower MDP models with factored state space and many followers - followers abstraction, structured dynamics and state aggregation. Frontiers in Artificial Intelligence and Applications.
158. Sanchez-Vazquez, MJ; Nielen, M; Edwards, SA; Gunn, GJ; Lewis, FI. 2012. Identifying associations between pig pathologies using a multi-dimensional machine learning methodology. BMC VETERINARY RESEARCH, doi:10.1186/1746-6148-8-151
159. Seetah, K; LaBeaud, D; Kumm, J; Grossi-Soyster, E; Anangwe, A; Barry, M. 2020. Archaeology and contemporary emerging zoonosis: A framework for predicting future Rift Valley fever virus outbreaks. INTERNATIONAL JOURNAL OF OSTEOARCHAEOLOGY, doi:10.1002/oa.2862
160. Shah, N; Malensek, M; Shah, H; Pallickara, S; Pallickara, SL. 2019. Scalable network analytics for characterization of outbreak influence in voluminous epidemiology datasets. CONCURRENCY AND COMPUTATION-PRACTICE & EXPERIENCE e4998, doi:10.1002/cpe.4998
161. Shah, N; Shah, H; Malensek, M; Pallickara, SL; Pallickara, S. 2016. Network Analysis for Identifying and Characterizing Disease Outbreak Influence from Voluminous Epidemiology Data. In: 4^th^ IEEE International Conference on Big Data (Big Data), Washington, DC
162. Sharifi, S; Pakdel, A; Ebrahimi, M; Reecy, JM; Farsani, SF; Ebrahimie, E. 2018. Integration of machine learning and meta-analysis identifies the transcriptomic bio-signature of mastitis disease in cattle. PLOS ONE e0191227, doi:10.1371/journal.pone.0191227
163. Simpraga, M; Smuc, T; Matanovic, K; Radin, L; Shek-Vugrovecki, A; Ljubicic, I; Vojta, A. 2013. Reference intervals for organically raised sheep: Effects of breed, location and season on hematological and biochemical parameters. SMALL RUMINANT RESEARCH, doi:10.1016/j.smallrumres.2012.11.032
164. Speybroeck, N; Lindsey, PJ; Billiouw, M; Madder, M; Lindsey, JK; Berkvens, DL. 2006. Modeling diapause termination of Rhipicephalus appendiculatus using statistical tools to detect sudden behavioral changes and time dependencies. ENVIRONMENTAL AND ECOLOGICAL STATISTICS, doi:10.1007/s10651-005-5691-1
165. Spiteri, M; Knowler, SP; Rusbridge, C; Wells, K. 2019. Using machine learning to understand neuromorphological change and image-based biomarker identification in Cavalier King Charles Spaniels with Chiari-like malformation-associated pain and syringomyelia. JOURNAL OF VETERINARY INTERNAL MEDICINE, doi:10.1111/jvim.15621
166. Tago D., Hammitt J.K., Thomas A., Raboisson D. 2016. The Impact of Farmers’ Strategic Behavior on the Spread of Animal Infectious Diseases. PLoS ONE 11(6): e0157450, doi:10.1371/journal.pone.0157450
167. Thulke H.-H., Lange M., Tratalos J.A., Clegg T.A., McGrath G., O’Grady L., O’Sullivan P., Doherty M.L., Graham D.A., More S.J. 2018. Eradicating BVD, reviewing Irish programme data and model predictions to support prospective decision making. Prev Vet Med 150, 151–161.
168. Tixier, P; Peyrard, N; Aubertot, JN; Gaba, S; Radoszycki, J; Caron-Lormier, G; Vinatier, F; Mollot, G; Sabbadin, R. 2013. Modelling Interaction Networks for Enhanced Ecosystem Services in Agroecosystems. Advances in Ecological Research, doi:10.1016/B978-0-12-420002-9.00007-X
169. Todorov, T; Stoinov, J. 2019. Expert System for Milk and Animal Monitoring. INTERNATIONAL JOURNAL OF ADVANCED COMPUTER SCIENCE AND APPLICATIONS, 10, 6, 25-30
170. Valdes-Donoso, P; VanderWaal, K; Jarvis, LS; Wayne, SR; Perez, AM. 2017. Using Machine learning to Predict swine Movements within a regional Program to improve control of infectious Diseases in the Us. FRONTIERS IN VETERINARY SCIENCE, doi:10.3389/fvets.2017.00002
171. VanderWaal, K; Morrison, RB; Neuhauser, C; Vilalta, C; Perez, AM. 2017. Translating Big Data into Smart Data for Veterinary Epidemiology. FRONTIERS IN VETERINARY SCIENCE, doi:10.3389/fvets.2017.00110
172. Vazquez-Diosdado, JA; Paul, V; Ellis, KA; Coates, D; Loomba, R; Kaler, J. 2019. A Combined Offline and Online Algorithm for Real-Time and Long-Term Classification of Sheep Behaviour: Novel Approach for Precision Livestock Farming. SENSORS, doi:10.3390/s19143201
173. Viet A-F., Krebs S., Rat-Aspert O., Jeanpierre L., Belloc C., Ezanno P. 2018. A modelling framework based on MDP to coordinate farmers' disease control decisions at a regional scale. PLOS ONE 13(6): e0197612. doi:10.1371/journal.pone.0197612
174. Viet, AF; Jeanpierre, L; Bouzid, M; Mouaddib, AI. 2012. Using Markov Decision Processes to define an adaptive strategy to control the spread of an animal disease. COMPUTERS AND ELECTRONICS IN AGRICULTURE, doi:10.1016/j.compag.2011.10.015
175. Vyas, S; Shukla, V; Doshi, N. 2019. FMD and Mastitis Disease Detection in Cows Using Internet of Things (IOT). In: 10^th^ International Conference on Emerging Ubiquitous Systems and Pervasive Networks (EUSPN) / 9^th^ International Conference on Current and Future Trends of Information and Communication Technologies in Healthcare (ICTH), Coimbra, PORTUGAL, doi:10.1016/j.procs.2019.11.019
176. Wagner, N; Antoine, V; Mialon, MM; Lardy, R; Silberberg, M; Koko, J; Veissier, I. 2020. Machine learning to detect behavioural anomalies in dairy cows under subacute ruminal acidosis. COMPUTERS AND ELECTRONICS IN AGRICULTURE, doi:10.1016/j.compag.2020.105233
177. Walsh, DP; Ma, TF; Ip, HS; Zhu, J. 2019. Artificial intelligence and avian influenza: Using machine learning to enhance active surveillance for avian influenza viruses. TRANSBOUNDARY AND EMERGING DISEASES, doi:10.1111/tbed.13318
178. Walsh, M; Haseeb, MA. 2015. Modeling the ecologic niche of plague in sylvan and domestic animal hosts to delineate sources of human exposure in the western United States. PEERJ e1493, doi:10.7717/peerj.1493
179. Walsh, MG; de Smalen, AW; Mor, SM. 2017. Wetlands, wild Bovidae species richness and sheep density delineate risk of Rift Valley fever outbreaks in the African continent and Arabian Peninsula. PLOS NEGLECTED TROPICAL DISEASES e0005756, doi:10.1371/journal.pntd.0005756
180. Walsh, MG; de Smalen, AW; Mor, SM. 2018. Climatic influence on anthrax suitability in warming northern latitudes. SCIENTIFIC REPORTS, doi:10.1038/s41598-018-27604-w
181. Walsh, MG; Mor, SM; Hossain, S. 2019. The elephant-livestock interface modulates anthrax suitability in India. PROCEEDINGS OF THE ROYAL SOCIETY B-BIOLOGICAL SCIENCES, doi:10.1098/rspb.2019.0179
182. Wang, L; Yao, DZ. 2013. A neural computational model for animal's time-to-collision estimation. NEUROREPORT, doi:10.1097/WNR.0b013e32835fa73f
183. Wang, T; Hennessy, DA. 2015. Strategic Interactions Among Private and Public Efforts When Preventing and Stamping Out a Highly Infectious Animal Disease. AMERICAN JOURNAL OF AGRICULTURAL ECONOMICS, doi:10.1093/ajae/aau119
184. Wardeh M, Sharkey KJ, Baylis M. 2020. Integration of shared-pathogen networks and machine learning reveals the key aspects of zoonoses and predicts mammalian reservoirs. Proc Biol Sci. 287(1920):20192882. doi: 10.1098/rspb.2019.2882.
185. Widgren S, Engblom S, Bauer P, Frössling J, Emanuelson U, Lindberg A. 2016. Data‑driven network modelling of disease transmission using complete population movement data: spread of VTEC O157 in Swedish cattle. Vet res 47:81
186. Widgren S, Engblom S, Emanuelson U, Lindberg A. 2018. Spatio‑temporal modelling of verotoxigenic Escherichia coli O157 in cattle in Sweden: exploring options for control. Vet Res 49:78
187. Williams, ML; James, WP; Rose, MT. 2017. Fixed-time data segmentation and behavior classification of pasture-based cattle: Enhancing performance using a hidden Markov model. COMPUTERS AND ELECTRONICS IN AGRICULTURE, doi:10.1016/j.compag.2017.11.010
188. Xu, X; Mazloom, R; Goligerdian, A; Staley, J; Amini, M; Wyckoff, GJ; Riviere, J; Jaberi-Douraki, M. 2019. Making Sense of Pharmacovigilance and Drug Adverse Event Reporting: Comparative Similarity Association Analysis Using AI Machine Learning Algorithms in Dogs and Cats. TOPICS IN COMPANION ANIMAL MEDICINE, doi:10.1016/j.tcam.2019.100366
189. Xu, ZY; Bagci, U; Mansoor, A; Kramer-Marek, G; Luna, B; Kubler, A; Dey, B; Foster, B; Papadakis, GZ; Camp, JV; Jonsson, CB; Bishai, WR; Jain, S; Udupa, JK; Mollura, DJ. 2015. Computer-aided pulmonary image analysis in small animal models. MEDICAL PHYSICS, doi:10.1118/1.4921618
190. Yan, XP; Li, J. 2019. Animal Intelligent Logistics Management Based on RFID Technology . REVISTA CIENTIFICA-FACULTAD DE CIENCIAS VETERINARIAS, 29, 6, 1772-1780
191. Yang, LH; Han, BA. 2018. Data-driven predictions and novel hypotheses about zoonotic tick vectors from the genus Ixodes. BMC ECOLOGY, doi:10.1186/s12898-018-0163-2
192. Yazdanbakhsh, O; Zhou, Y; Dick, S. 2017. An intelligent system for livestock disease surveillance. INFORMATION SCIENCES, doi:10.1016/j.ins.2016.10.026
193. Yeganejou, M; Dick, S. 2017. Inductive Learning of Classifiers via Complex Fuzzy Sets and Logic. In: IEEE International Conference on Fuzzy Systems (FUZZ-IEEE), Naples, ITALY
194. Yousefi Naghani S, Dara R, Poljak Z, Sharif S. 2019. A review of knowledge discovery process in control and mitigation of avian influenza. Anim Health Res Rev 20(1):61-71. doi: 10.1017/S1466252319000033
195. Zenger, KR; Khatkar, MS; Jones, DB; Khalilisamani, N; Jerry, DR; Raadsma, HW. 2019. Genomic Selection in Aquaculture: Application, Limitations and Opportunities With Special Reference to Marine Shrimp and Pearl Oysters. FRONTIERS IN GENETICS, doi:10.3389/fgene.2018.00693
196. Zhang, XW; Yang, RQ; Cheng, CR. 2004. Design and application of animal shed environment intellectual control system. In: PROCEEDINGS OF THE WORLD ENGINEERS' CONVENTION 2004, VOL E, AGRICULTURAL ENGINEERING AND FOOD SECURITY, Shanghai, CHINA.
197. Zhuang, XL; Bi, MN; Guo, JL; Wu, SY; Zhang, TM. 2018. Development of an early warning algorithm to detect sick broilers. COMPUTERS AND ELECTRONICS IN AGRICULTURE, doi:10.1016/j.compag.2017.11.032

**B - Interviews**

All units (19) of the Animal Health Division (AHD) of INRAE, France, were contacted. Persons were targeted on the advice of unit heads and members of the working group. Additional interviews were conducted in other divisions of INRAE and outside INRAE, to get a view of the forces and skills present at the IA/AH interface, the interest of scientists in this interface, and illustrations of groups organised at a comparable interface (in the field of health *s.l.*).

- INRAE, Santé Animale (Animal health division): C. Citti (Toulouse), I. Oswald & P. Martin (Toulouse), D. Concordet & A. Bousquet-Mélou (Toulouse), S. Picault, G. Beaunée, A. Madouasse & P. Ezanno (Nantes), CaSciModOT project (Val-de-Loire region)
- INRAE, other divisions:
  - MATHNUM (Mathematics and digital technologies): H. Monod (Head of the Division, Ile-de-France-Jouy-en-Josas), F. Garcia & T. Schiex (Toulouse), E. Vergu (Jouy-en-Josas)
  - SPE (Plant health and environment): C. Lannou (Head of the Division, Ile-de-France-Jouy-en-Josas)
  - ECODIV (Ecology and biodiversity of forest, grassland and freshwater environments): C. Bastien (Head of the Division, Grand-Est-Nancy), A. Franc (Bordeaux)
  - ACT (Action and transitions): Charline Smadi (Grenoble), Maguelonne Teisseire (Montpellier)
- Inserm/Inria: R. Thiébaut (Bordeaux).

The interviews covered the following points:

- What research is being conducted in the team(s) related to AI? What added-value for AH research? What is the position in relation to the list of themes selected?
- Is this research collaborative? What key skills are mobilised?
- What is the main lock? (e.g., data, skills, methods, collaborations)
- What science front at this IA/AH interface? Positioned in AH (new questions can be addressed or questions revisited) or in AI (development of new methods, new concepts)?
- What concrete example to illustrate the point?
- Who else should we contact?

The IA themes selected were as follows:

- Knowledge representation: domain-specific language, ontology
- Symbolic or numerical meta-modelling, automatic computer code generation, autonomous code adaptation
- Simulation architecture, multi-agent multi-level systems, large / multi-scale
- Problem solving:
  - Multi-criteria optimisation, reasoned exploration of scenarios to automatically identify best scenarios;
  - Allocation of resources under constraints: IT resources, resources internal to the modelled system;
  - Machine Learning: Bayesian networks, neural networks, supervised or unsupervised classification methods; pattern recognition, image analysis; data mining;
- Decision: development of adaptive strategies, game theory, Markovian approaches, explicit coupling of epidemiological models and economic decision models.

**C – Previous publication in French**

For a complete information of the readers, a previous manuscript but substantially different from this English version has already been accepted for publication in French in *INRAE Productions Animales*. The associated reference is:

Ezanno P, Picault S, Winter N, Beaunée G, Monod H, Guégan J-F (2020) Intelligence artificielle et santé animale. INRAE Productions Animales 33:95-108, <https://doi.org/10.20870/productions-animales.2020.33.2.3572>.
